# Assessing the Impact of a Novel Smartphone Application Compared With Standard Follow-Up on Mobility of Patients With Knee Osteoarthritis Following Treatment With Hylan G-F 20: A Randomized Controlled Trial

**DOI:** 10.2196/mhealth.7179

**Published:** 2017-05-09

**Authors:** Nebojsa Skrepnik, Andrew Spitzer, Roy Altman, John Hoekstra, John Stewart, Richard Toselli

**Affiliations:** ^1^Tucson Orthopaedic InstituteTucson, AZUnited States; ^2^Cedar-Sinai Orthopaedic CenterLos Angeles, CAUnited States; ^3^University of California Los Angeles Medical CenterLos Angeles, CAUnited States; ^4^National Clinical Research–RichmondRichmond, VAUnited States; ^5^SanofiLaval, QCCanada; ^6^SanofiParisFrance

**Keywords:** mobile health, mHealth, mobile apps, osteoarthritis, osteoarthritis, knee, hylan G-F 20, Synvisc

## Abstract

**Background:**

Osteoarthritis (OA) is a leading cause of disability in the United States. Although no disease-modifying therapies exist, patients with knee OA who increase walking may reduce risk of functional limitations.

**Objective:**

The objective of the study is to evaluate the impact of a mobile app (OA GO) plus wearable activity monitor/pedometer (Jawbone UP 24) used for 90 days on the mobility of patients with knee OA treated with hylan G-F 20.

**Methods:**

Patients with knee OA aged 30 to 80 years who were eligible to receive hylan G-F 20 and were familiar with smartphone technology were enrolled in this randomized, multicenter, open-label study. Patients who had a body mass index above 35 kg/m^2^ were excluded. All patients received a single 6-mL injection of hylan G-F 20 and wore the Jawbone monitor. The patients were then randomized 1:1 to Jawbone and OA GO (Group A; n=107) with visible feedback (unblinded) or Jawbone only (Group B; n=104) with no visible feedback (blinded). The primary endpoint was mean change from baseline in steps per day at day 90 between Groups A and B.

**Results:**

Baseline characteristics were similar between groups. There were significant differences between the increases in least squares (LS) mean number of steps per day (1199 vs 467, *P*=.03) and the mean percentage change (35.8% vs 11.5%, *P*=.02) from baseline in favor of Group A over Group B. There was a greater reduction in pain from baseline during the 6-minute walk test in Group A versus Group B. (LS mean change: −55.3 vs −33.8, *P*=.007). Most patients (65.4%) and surveys of physicians (67.3%) reported they would be likely or very likely to use/recommend the devices. Patient Activity Measure-13 scores improved from baseline (LS mean change for Groups A and B: 5.0 vs 6.9), with no significant differences between groups. The occurrence of adverse events was similar in the 2 groups.

**Conclusions:**

Use of a novel smartphone app in conjunction with a wearable activity monitor provided additional improvement on mobility parameters such as steps per day and pain with walking in the 6-minute walk test in patients with knee OA who were treated with hylan G-F 20. Results also highlight the amenability of patients and physicians to using mobile health technology in the treatment of OA and suggest further study is warranted.

## Introduction

### Osteoarthritis

Osteoarthritis (OA) is a leading cause of disability in the United States [1]. From 2010 to 2012, an estimated 52.5 million (22.7%) US adults reported doctor-diagnosed arthritis, representing a net increase of 0.87 million adults with arthritis per year since the 2007 to 2009 estimate of 49.9 million [2]. The prevalence of OA, the most common form of arthritis [1], is estimated to double by 2020 [3] as predicted from the increase in the number of US adults with clinical hand, hip, or knee OA from 21 million in 1995 to 27 million in 2005 [4]. Estimates suggest that symptomatic knee OA occurs in 13% of women and 10% of men aged 60 years and older [5], approximately 17% of adults aged 45 years and older [4], and approximately 5% of adults aged 26 years and older [4]. Furthermore, the number of people affected by symptomatic OA is likely to increase as the population ages and the rate of obesity increases [5]. OA of the knee can significantly contribute to pain and lack of physical activity [2,6,7]. The resulting reduction in mobility has also been found to negatively impact quality of life [8] and result in an increased risk of sick leave and need for disability [9]. A study using US national survey data found that OA-associated absenteeism costs equaled approximately 3 lost work days per year, totaling up to $10.3 billion in annual absenteeism costs [10].

### Management of Knee Osteoarthritis

To date, there is no disease-modifying therapy available for OA [11]. Optimal management of knee OA requires a combination of both pharmacologic (eg, oral or topical analgesics or intra-articular therapies [viscosupplementation, corticosteroids]) and nonpharmacologic methods (eg, weight loss, exercise, and physical activity) [12-15]. However, despite the well-recognized benefits of exercise and the promotion of physical activity by many professional societies [12,13,15], there is no standard exercise or education program and no clear benefit of one exercise program over another [13]. Information about the benefits of exercise is readily available [12,13,15] but rarely incorporated into patient behavior [16,17], and adherence to such programs by patients with knee OA is poor [16,17]. There is also no consensus on which measures or combination of measures should be used to assess physical function in patients with knee OA [18], complicating the initial assessment and tracking of progress in mobility improvement.

Walking has been shown to significantly reduce symptoms and the risk of functional limitations due to knee OA [19,20]. In one study, patients with OA of the knee who walked 6,000 steps per day or more reduced the risk of developing a functional limitation by half within the next 2 years [19]. This suggests that walking may maintain knee function. However, strategies to encourage adherence to regular walking are clearly needed.

### Benefits of Mobile Health Apps

Adoption of mobile health apps has the potential to improve patient outcomes. Mobile health apps have been shown to be a useful tool in weight loss programs [21,22]. In addition, the use of short message service text message reminders has been shown to improve health care appointment attendance across health care settings [23], and there is evidence from a randomized, controlled trial to suggest that monthly phone contact can result in clinical improvements in patients with knee OA [24]. Patient physical activity level has been found to increase in studies using Internet- and mobile-based apps for postrehabilitation exercise persistence in chronic obstructive pulmonary disease [25], in physical activity maintenance following cardiac rehabilitation [26], in regular physical activity engagement by cancer survivors [27], in increasing walking based in the workplace [28], and in promoting a healthy lifestyle [29].

The use of mobile apps in health care may be one such strategy to increase physical activity and enhance OA management. However, there are currently no published data on the use of mobile apps for the management of knee OA.

The objective of this study was to examine the impact on mobility in knee OA patients who were treated with hylan G-F 20 and standard of care follow-up plus a mobile smartphone app (OA GO) and a wearable activity monitor (Jawbone UP 24) versus hylan G-F 20 and standard of care follow-up only over 90 days. We hypothesized that patients using the OA GO app and a wearable activity monitor would experience a positive impact on their mobility compared with those not using the app.

## Methods

### Study Population

Consecutive patients with knee OA whom the physician investigator decided to treat with one 6-mL injection of hylan G-F 20 (in accordance with the US label) who consented and qualified were enrolled in this open-label, multicenter, randomized, parallel-group study. Study sites and physicians were recruited using a detailed questionnaire and site qualification visit. Patients were recruited from the selected private community-based practices and research-only practice sites. All eligible patients were able to read and understand English and provided informed consent prior to starting the study. The protocol complied with the 18th World Health Congress (Helsinki, 1964) recommendations and applicable amendments and with any applicable country-specific laws, regulations, and guidelines. The protocol was approved by relevant ethics committees and/or institutional review boards. After working with the US Food and Drug Administration (FDA) to initiate registration of the trial, the sponsor deemed the study not eligible to be posted on ClinicalTrials.gov considering that clinical studies using devices whereby the primary outcome measure relates solely to feasibility and not to health outcomes are excluded from registration. The FDA concurred there was not a requirement to register the trial at that time.

To be included in the study, all patients must have had unilateral knee OA and have been suitable for treatment with hylan G-F 20 based on the decision of the physician investigator. Study sites were requested to report whether or not a knee examination was performed or x-ray was taken but the results of these examinations were not captured.

Patients were excluded if they were aged younger than 30 years or older than 80 years, were unfamiliar with smartphones, or had baseline pain greater than 9 on the 11-point numeric pain rating scale (NPRS; pain ratings could range from 0, no pain, to 5, moderate pain, to 10, worst possible pain) in the target-for-treatment knee while walking on a flat surface. Patients with bilateral disease were excluded with the exception of patients who were treated in only one knee and had contralateral knee pain less than 4 on NPRS while walking on a flat surface. Patients whose baseline daily step average was less than 500 or more than 8000 as assessed during the screening and run-in phases were not eligible. Also excluded were patients who had a body mass index (BMI) greater than 35 or life expectancy less than 12 months, were currently using a wearable activity monitor or analogous device, had planned surgery on any lower extremity joint or any significant medical condition that would interfere with study participation, were chronic narcotic users, or were pregnant or breastfeeding or likely to become pregnant. The protocol did not specifically exclude patients based on use of previous intra-articular injections (cortisone or hyaluronic acid).

### Study Design

All eligible patients received hylan G-F 20. Participants wore a commercially available activity monitor (Jawbone UP 24) on their wrists and were instructed to remove the monitor only during the weekly charging times and in situations where the device would be submerged in water. Patients were randomized 1:1 (stratified by site) to Group A or Group B. The randomization scheme was generated by the study sponsor and stratified by site. Sealed envelopes, numbered in an ascending order for use, were provided to each site. The envelopes were opened according to ascending sequence to ensure proper randomization.

**Figure 1:**
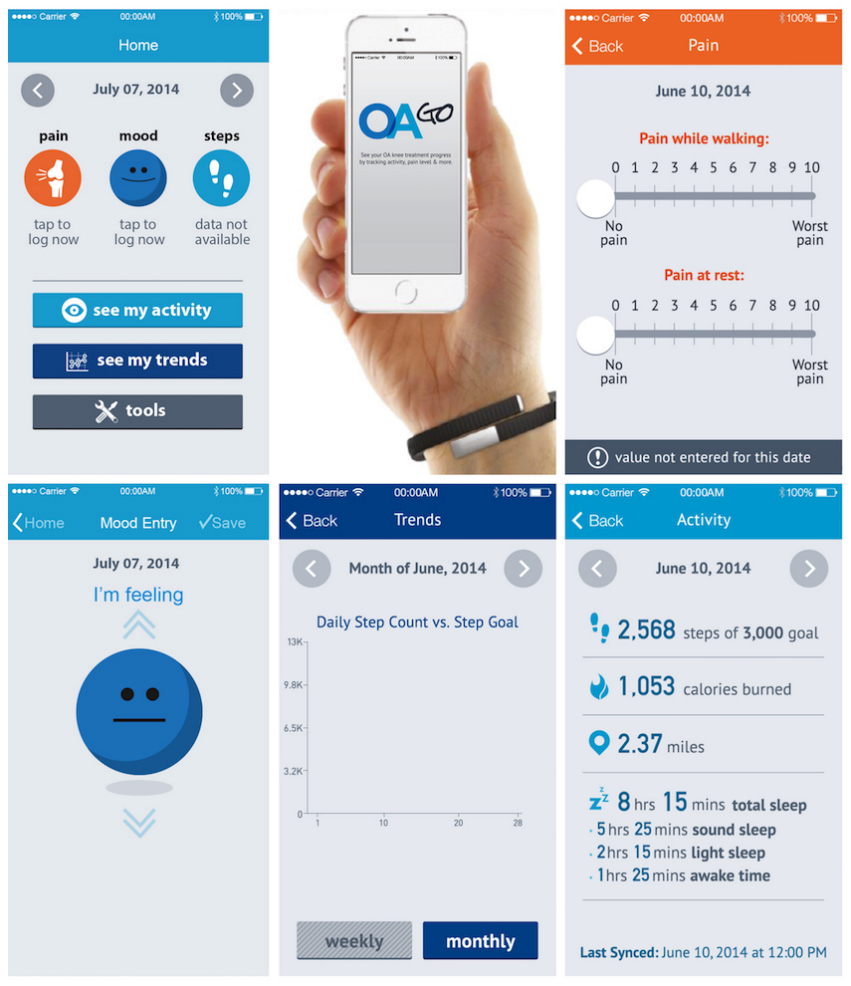
OA GO mobile app.

Group A patients were provided with regular follow-up as per standard-of-care (information on the benefits of walking in a brochure available from the Arthritis Foundation) plus an unblinded wearable activity monitor and a mobile app (OA GO) (Figure 1). The OA GO app (downloaded to a trial-sponsored iPhone 5 or newer) provided motivational messages and requested that the patient enter pain and mood data on a once-daily basis. Trial coordinators demonstrated app use, provided charging instructions for the Jawbone UP 24, and set the daily step goal based on patient’s baseline steps per day during screening. The OA GO app combined continuously retrieved data from the wearable activity monitor with data entered by the patient to display the patient’s daily step count, calories burned, and sleep. Daily and monthly cumulative activity trends were available for the patient to review. Group B was provided with regular follow-up plus a blinded wearable activity monitor, with instructions to wear the monitor at all times except during weekly charging and water activities. The patients in Group B received standard-of-care instructions and education but did not have access to activity recorded by the wearable activity monitor. In this group, data from the activity monitor were downloaded by the study team at last visit. The study consisted of 5 visits: screening and baseline (days −7 and 1) with follow-up visits at days 7 and 30, and day 90, last visit. The main study duration was 90 days. A poststudy adherence check in Group A patients occurred at day 180, when data were downloaded from the app (no visit).

### Assessments

The primary endpoint was mean change from baseline to day 90 in mobility as measured by steps per day. Baseline steps per day was the average over at least 3 days, and last assessment was the average steps per day over the 7 days immediately preceding the last visit. Change was calculated as the difference between the 2 averages. Use of average steps per day avoided bias due to any one particularly good or bad day for the patient. The secondary endpoints were mean percentage change from baseline in steps per day at each assessment visit (average of a 7-day period), and at day 90, mean percentage change from baseline in the 6-minute walk test (distance and pain assessed by the NPRS), patient and physician satisfaction with treatment, percentage change in Patient Activation Measure (PAM)-13 questionnaire score [30], percentage change in sleep captured by the wearable activity monitor (light, sound, and duration of sleep), and Visual Analog Mood Scale (VAMS) assessment. Treatment-emergent adverse events (TEAEs) were also assessed.

For Group A, the satisfaction of patients and physicians was captured using survey questionnaires. The patient survey consisted of 7 questions: the first 6 were answered on a 7-point Likert scale related to the extent to which the patients experienced changes (−3 to +3, where 0=no change), and the seventh was a 4-point scale asking about their likelihood of using the device in the future (not at all likely to very likely). Valid questionnaires were those in which at least 4 of the first 6 questions were answered. A physician survey was similarly constructed with a final question, using the 4-point scale, which asked whether they would recommend the device in the future (not likely to very likely). Physicians answered the survey at day 90, before viewing the data, and must have answered at least 3 of the first 4 questions. All responses to survey questions were based on patient and physician observations and opinions. The survey questionnaires were developed for this study and were not externally validated.

The PAM-13 [30] was used to assess patients’ knowledge, skills, and confidence for self-management at randomization and last visit, with responses given on a Guttman-like scale that ranged from 1 to 4 (strongly disagree to strongly agree). Unanswered items were scored as missing. Raw scores ranged from 13 to 52, with higher scores indicating more activation; raw scores were converted to activation scores, which ranged from 0 to 100, with a score of 100 corresponding to the highest degree of activation. The PAM-13 is a reliable and valid measure of patient activation with higher activation scores associated with increased self-management behaviors and increased self-efficacy with respect to patients taking charge of their overall health and involvement in their care. As a result, the concept of activation can be useful for evaluating interventions and for tailoring care plans for individual patients in a clinical setting [30,31].

The VAMS was used to assess mood as captured in 8 domains with raw scores transformed into T-scores with a mean of 50 and standard deviation of 10. The VAMS has been validated in both normal and neurologically impaired individuals as a brief measure of internal mood state [32,33].

### Statistical Analysis

The Consolidated Standards of Reporting Trials guidelines were followed [34]. A sample size of 200 randomized (172 evaluable) patients (100 randomized [86 evaluable] per group) was estimated to provide 80% power to detect an average increase in the change from baseline steps per day of 25% for Group A compared with Group B, assuming a 2-sided significance level of 5%. The variability was expected to be similar in the 2 groups, and an estimate of 58% for the coefficient of variation was used in this calculation [35]. The sample size of 200 randomized patients assumed a drop-out rate of 15%.

All efficacy endpoints were analyzed in the modified intent-to-treat population, which included all randomized patients who had both a baseline value and an on-treatment value for the primary endpoint on or after day 30. All efficacy endpoint data comparing change from baseline between groups were obtained from an analysis of covariance model with baseline mean steps per day as the covariate and treatment assignment and pooled site as class variables. They are presented as least squares (LS) means with 95% confidence intervals (CIs). Statistical analyses were based on initial verification of parametric model assumptions, and a rank analysis of covariance was then considered if conditions for a parametric model were not met. To allay concerns by the readers, nonparametric analyses are presented here. Patient and physician satisfaction surveys at last visit were summarized.

Safety endpoints were analyzed for all patients provided with a wearable activity monitor. Adverse events (AEs) were presented as the number and percentage of patients experiencing an AE. Multiple occurrences of the same event in the same patient were counted only once. The denominator for computation of percentages is the safety population within each randomized group.

## Results

### Patient Disposition, Baseline Characteristics, and Adherence

**Figure 2:**
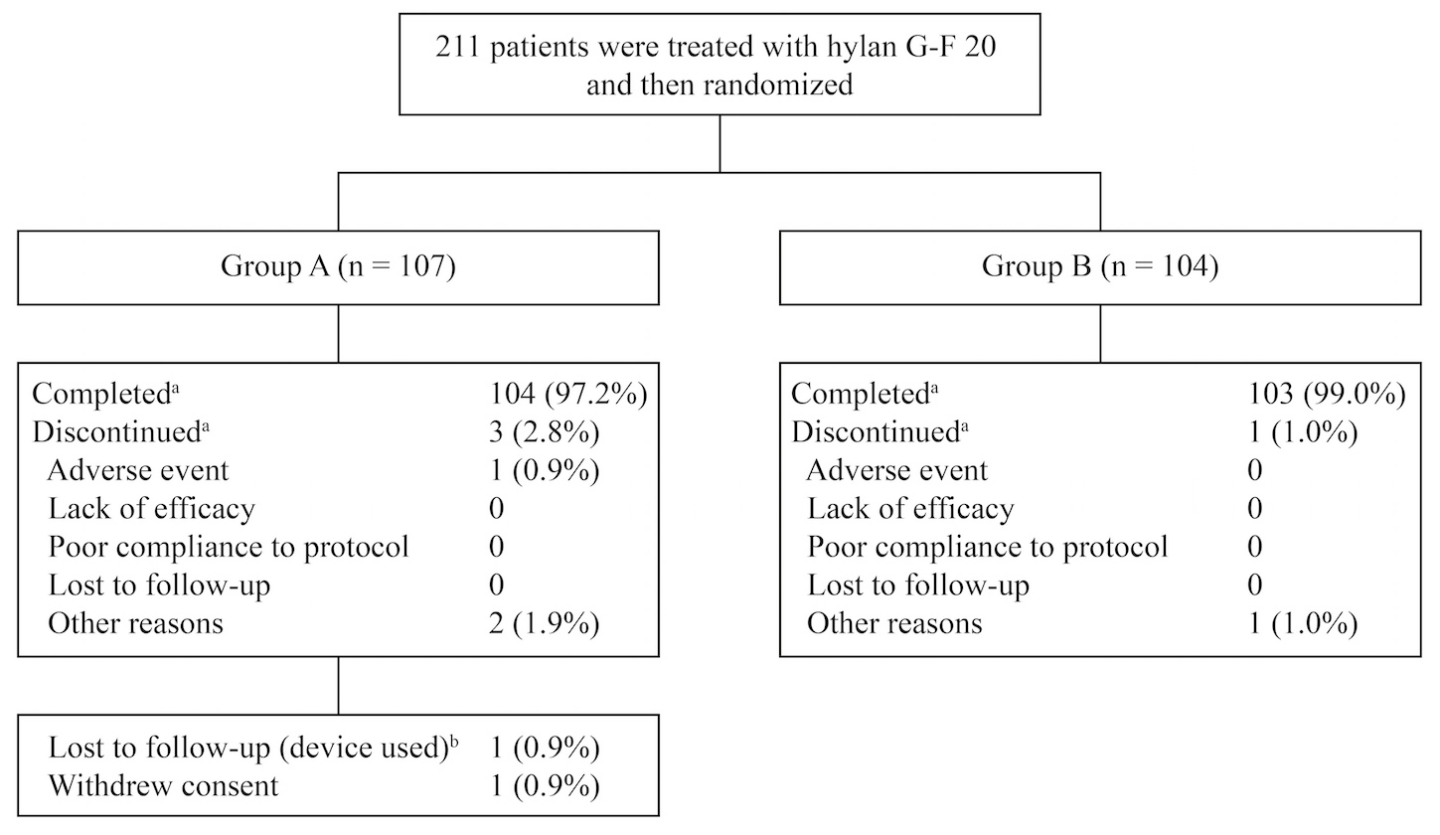
Patient disposition (a: 90-day observation period, b: 91 to 180 days).

All patients had a knee exam at baseline, and 119 of 211 (56.4%) had an x-ray of the knee. A total of 211 knee OA patients treated with hylan G-F 20 were randomized (Group A=107; Group B=104), and nearly all patients completed the 90-day observation period (Group A, 104/107 [97.2%]; Group B, 103/104 [99.0%]) (Figure 2). Almost all Group A patients (101/107) decided to continue using the OA GO app and entered the 90 to 180 days adherence period, and 81/101 (80.2%) of these patients completed the 180 days. Baseline characteristics were similar between the 2 groups (Table 1) with a mean patient age of 62.6 (SD 9.4) years. For the total population at baseline, mean number of steps per day was 4276 (SD 1807) and pain NPRS score on the 6-minute walk test was 4.8 (SD 2.2), with 127/211 (60.2%) patients reporting pain of 5 or greater. Baseline PAM-13 score for the total population was 71.2 (SD 13.2), indicating that patients were highly activated. Overall, 91.0% of patients were compliant with the activity monitor (defined as using the wearable activity monitor 80% or more of the time). Group A had 103/107 (96.3%) patients and Group B had 80/104 (76.9%) patients who were compliant. Of the patients in Group A who entered the 90 to 180 days adherence period, 36/101 (35.6%) were 80% or more compliant with use of the OA GO app.

**Table 1:** Patient demographics and baseline characteristics.

		Group A (n=107)	Group B (n=104)	Total (N=211)
**Characteristic**	
	Age (years), mean (SD)	61.6 (9.5)	63.6 (9.3)	62.6 (9.4)
	Male, n (%)	48 (44.9)	57 (54.8)	105 (49.8)
	Caucasian, n (%)	87 (81.3)	98 (94.2)	185 (87.7)
	BMI^a^, kg/m^2^, mean (SD)	29.4 (3.9)	29.3 (3.4)	29.3 (3.7)
	Steps per day, mean (SD)	4279.7 (1787.3)	4271.5 (1837.0)	4275.7 (1807.2)
**6-minute Walk Test**	
	Pain NPRS^b^, mean (SD)	4.6 (2.3)	5.1 (2.0)	4.8 (2.2)
	Distance, meters, mean (SD)	402.8 (120.5)	395.6 (104.2)	399.3 (112.6)
**PAM-13^c^**	
	Activation score, mean (SD)	71.7 (13.5)	70.6 (13.0)	71.2 (13.2)

^a^BMI: body mass index.

^b^NPRS: numeric pain rating scale.

^c^PAM-13: Patient Activation Measure-13.

### Mobility and Pain

**Figure 3:**
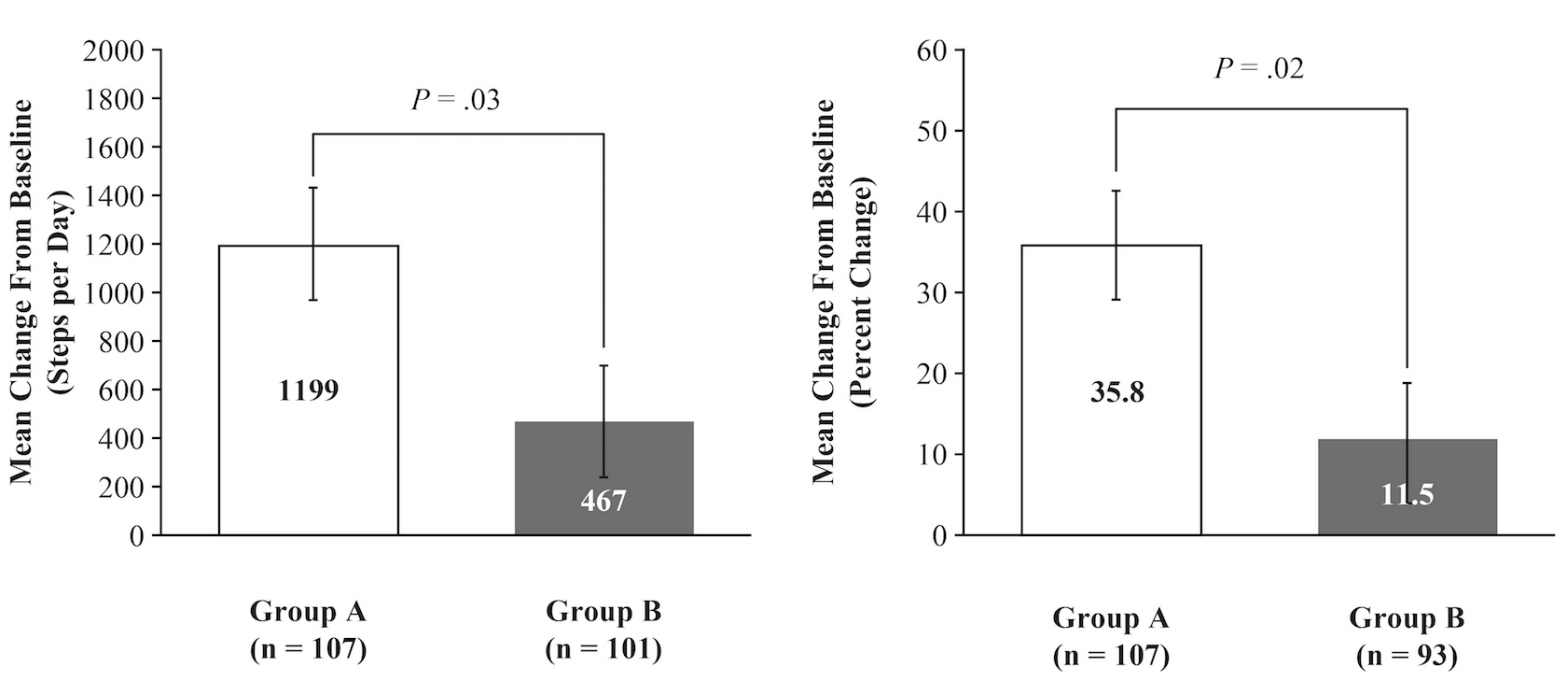
Steps per day. Mean change from baseline at day 90 in number and mean percentage change from baseline. Data are presented as least squares means and standard error. P values obtained from rank analysis of covariance.

A significant increase from baseline to last assessment in mean number of steps per day was observed for all subjects (916 [SE 14.4]; *P*<.001). Improvement in mobility at day 90 was significantly greater in Group A than in Group B. LS mean change in number of steps per day was 1199 vs 467, a mean difference of 732 steps (95% CI 127-1337; *P*=.03) (Figure 3).

An increase in mean steps per day was observed for both groups at each visit. The percentage increase in steps per day was significantly greater for all visits in Group A versus Group B. LS mean percentage change in steps per day for Group A versus Group B at day 7 was 16.8% versus 3.1% (mean difference: 13.7%, 95% CI 0.7-26.7; *P*=.03), at day 30 was 40.1% versus 9.0% (mean difference: 31.1%, 95% CI 16.2-45.9; *P*<.001), and at day 90 was 35.8% versus 11.5% (mean difference: 24.3%, 95% CI 6.9-41.7; *P*=.02) (Figure 3).

**Figure 4:**
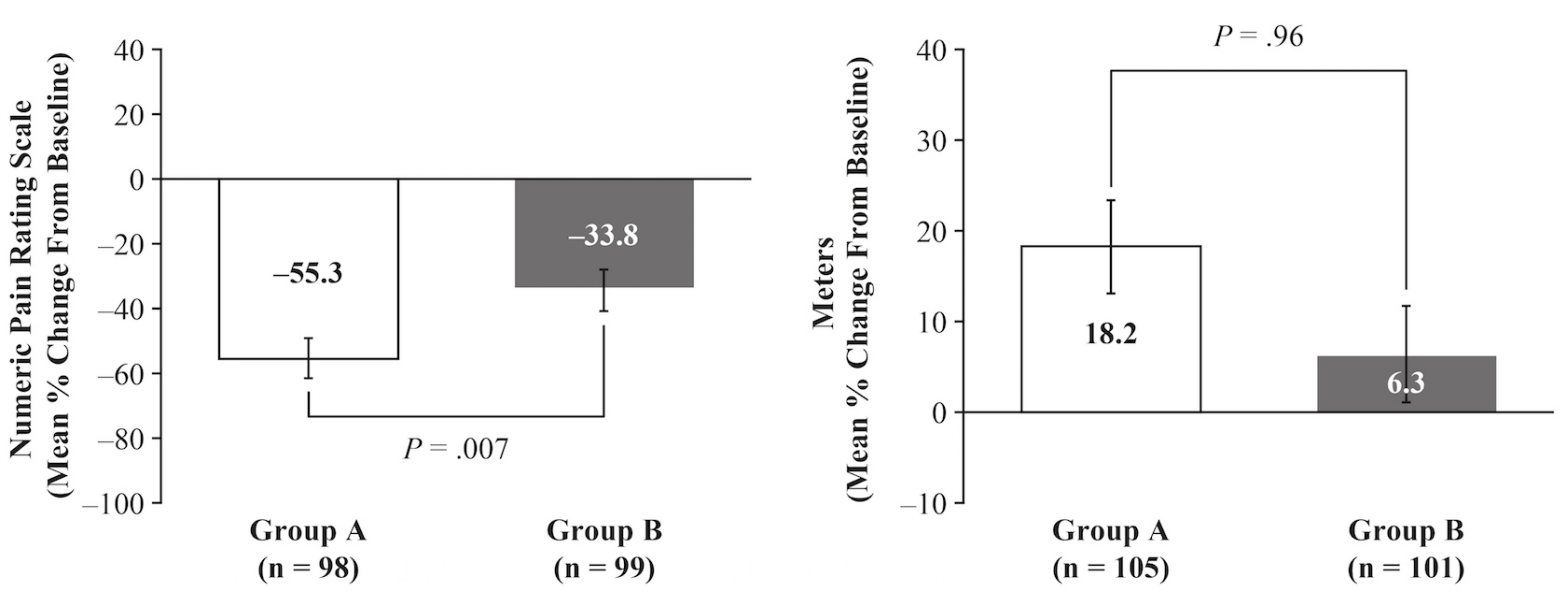
Six-minute walk test. Mean percentage change from baseline to day 90 in pain during the test and distance walked. Data are presented as least squares means and standard error. P values obtained from rank analysis of covariance.

In the 6-minute walk test, Group A experienced a significantly greater improvement from baseline to day 90 in LS mean percentage change in pain versus Group B, −55.3% versus −33.8% (mean difference: −21.5%, 95% CI −37.8 to −5.2; *P*=.007) (Figure 4). There was also a numerical, but not a statistically significant, improvement in LS mean percentage change in distance in the 6-minute walk test for Group A versus Group B of 18.2% versus 6.3% (mean difference: 11.9%, 95% CI −1.4 to 25.1; *P*=.96) (Figure 4).

### Quality of Life

**Figure 5:**
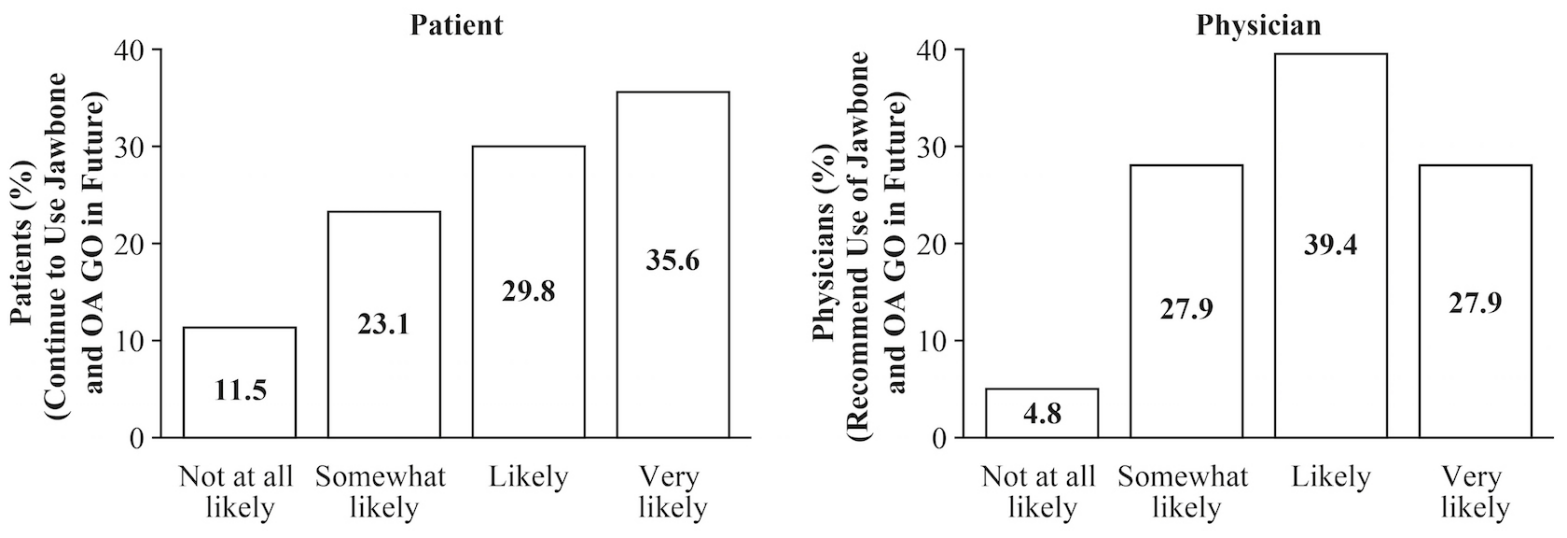
Satisfaction survey results in patients and physicians. Only patients in Group A (n=104) and their associated physicians participated in the satisfaction survey, which was completed at day 90.

A greater number of Group A patients (68/104, 65.4%) reported they would be likely or very likely to use the devices compared with patients (36/104, 34.6%) who reported that they would be somewhat likely or not at all likely to do so (Figure 5). A total of 76 physicians answered surveys for 104 patients; 70 of 104 (67.3%) physician surveys reported physicians to be likely or very likely to recommend use of the devices versus 34 (32.7%) surveys reporting physicians to be only somewhat likely or not at all likely to recommend their use (Figure 5).

**Figure 6:**
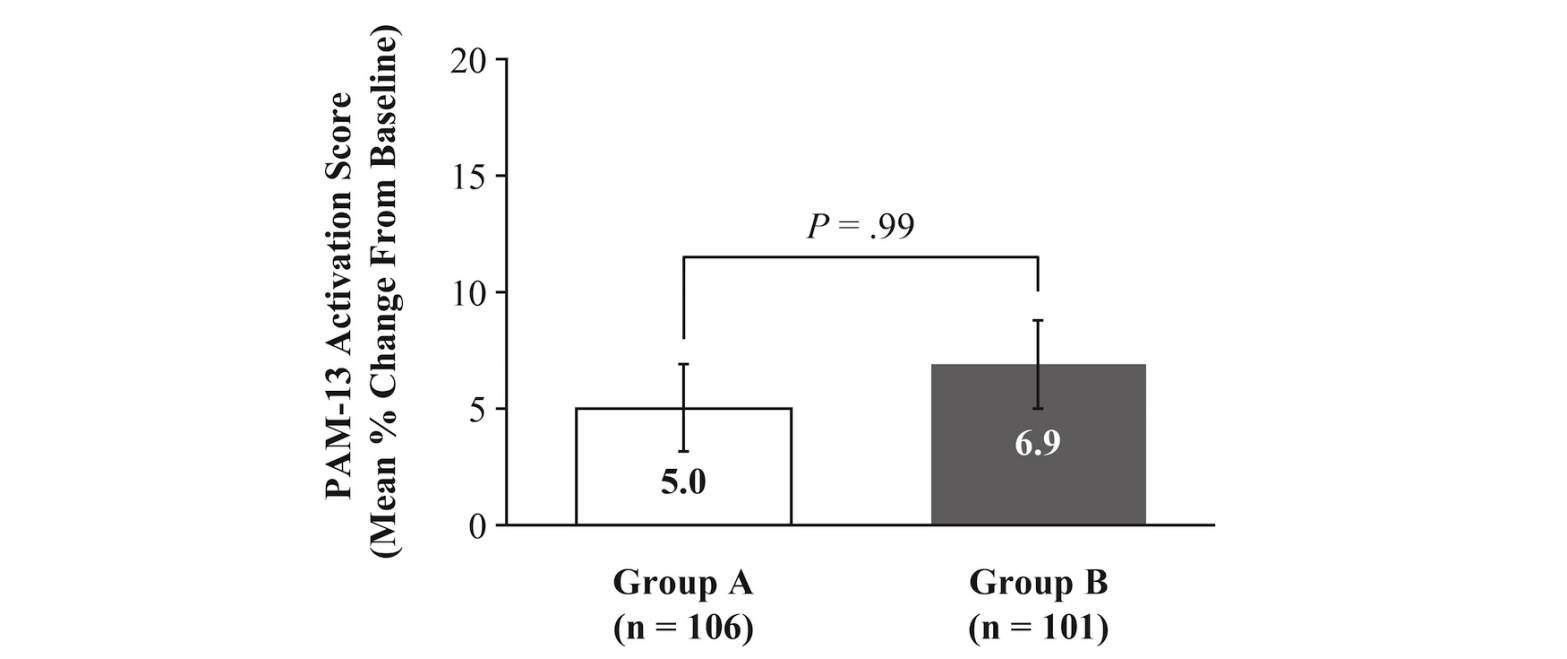
Mean change from baseline in Patient Activation Measure-13. Data are presented as least squares means and standard error.

PAM-13 scores improved from baseline to day 90 in both groups. The LS mean change from baseline was 5.0% in Group A versus 6.9% in Group B (Figure 6; mean difference –1.9%, 95% CI –6.8% to 3.1%; *P*=.99).

There were no significant changes in sleep from baseline to day 90 for either group and no significant differences between groups. Changes in VAMS scores from baseline to day 90 were also not significantly different between groups.

### Safety and Tolerability

The occurrence of TEAEs was similar in the 2 groups (Table 2) with no new safety signals noted. Arthralgia (Group A, 8/107 [7.5%]; Group B, 12/104 [11.5%]) and upper respiratory tract infection (Group A, 7/107 [6.5%]; Group B, 2/104 [1.9%]) were the 2 most commonly reported TEAEs among patients. No major AEs or treatment-emergent serious AEs related to the device or protocol occurred.

**Table 2:** Treatment-emergent adverse events.

		Group A (n=107)	Group B (n=104)
Any TEAE^a,b^		42 (39.3)	38 (36.5)
**Any TEAE occurring in ≥2% of patients**			
	Arthralgia	8 (7.5)	12 (11.5)
	Upper respiratory tract infection	7 (6.5)	2 (1.9)
Any serious TEAEs^c^		5 (4.7)	1 (1.0)
Any TEAE leading to death		0	0
Any TEAE leading to discontinuation of Jawbone^d^		1 (0.9)	0
Any TEAE leading to discontinuation of OA GO^d,e^		1 (0.9)	—

^a^TEAE: treatment-emergent adverse events.

^b^All TEAEs were mild to moderate in severity.

^c^Serious AEs included upper respiratory tract infection, transient ischemic attack, large intestine perforation, arthralgia and worsening OA, each in one patient in Group A and cholecystitis in one patient in Group B.

^d^Same patient discontinued both devices; the TEAE leading to discontinuation was worsening OA.

^e^OA: osteoarthritis.

## Discussion

### Principal Findings

Walking has been shown to have a beneficial effect on symptoms and decreases risk of functional limitations in patients with knee OA [19,20]; however, 66% of patients with arthritis reported walking for fewer than 90 minutes per week [36]. This study showed that, in patients treated with hylan G-F 20, those also using the OA GO app significantly improved their mobility with an increase in over twice as many steps per day and over 3 times the percentage change in steps per day compared with patients not using this motivating app. The increased mobility experienced by the patients using OA GO was also accompanied by a significantly greater reduction in reported pain compared with that for those not using the app. In addition, most patients and physicians expressed satisfaction with the use of the app and wearable activity monitor, suggesting that they would be amenable to the adoption of the technology in their clinical practice and daily lives. The above findings as a whole are clinically and socially important, given the recognition that increased mobility is closely associated with improved quality of life [8]. This study is the first to demonstrate that the use of a motivating mobile app can increase mobility and improve pain outcomes in patients with knee OA.

### Implication of Study Findings

Use of the OA GO app provides direct feedback on mobility function and pain (whereby steps and mobility become surrogates for pain and function, assuming there are no other reasons for decreased activity), which should give a more valid and reliable report of pain over time as it does not rely on patient recall of pain experienced. In contrast, visual analog scales [37] and the Western Ontario and McMaster Universities Osteoarthritis Index (used to assess pain, stiffness, and physical function over a specific time frame [ie, the last 48 hours] in clinical trials [38]) are both subjective measures that rely on patients thinking back to how they perceived they were feeling. Furthermore, symptoms of OA are often variable depending on a number of factors, including patients’ activity level [7] and changes in the weather [39], suggesting that pain should be assessed in the context of ongoing physical activity levels. This technology, therefore, is an objective measure of functional improvement over time resulting from clinical interventions provided to patients, making it not only a motivating device for patients but also an important research tool.

In this study, the results of the distance portion of the 6-minute walk test were not statistically different between groups. However, this assessment represents only a very brief snapshot in time; distance monitored continuously may be a more reliable measure of improvement in patient mobility. Despite these results not achieving statistical significance, those using the OA GO app ended the study with an average of more than 5500 steps per day, which approaches 6000 steps per day, a level that has been shown to cut the risk of developing functional limitation by half within 24 months [19].

PAM-13 scores were also not statistically different between groups in this study. The observed lack of statistical difference between groups could be due to the high mean baseline activation state of patients in this study (71.2 [SD 13.2]), which may have resulted from the selection criteria (ie, selecting relatively high functioning patients with knee OA) and may have been higher than for those excluded from the study. Indeed, the baseline activation score in this study was higher than scores for the general population in the US (61.9 [SD 21]) and populations with other chronic conditions (a US population with diabetes and with/without other comorbidities, 57.1 [40]; a Korean OA population, 56.0 [41]; and a Norwegian community mental health center population, 51.9 [42]). Evidence suggests that patients who start at the lowest activation levels tend to increase the most, suggesting that patients in this study may have been approaching a ceiling at which their ability to further increase activation levels may have been reduced, compared with patients whose baseline activation levels were lower [43].

### Limitations

This study does have some limitations. The patients studied may not be fully representative of the general OA population with respect to gender distribution (women are at higher risk for knee OA than men [44]), and activity levels used as inclusion and exclusion criteria were such that patients with low levels (less than 500 steps per day) and high levels (more than 8000 steps per day) of baseline activity were not eligible for enrollment. Patients with a BMI above 35 were also excluded from the study. In addition, the Kellgren-Lawrence grade of patients’ knee OA was not collected, so data could not be stratified and analyzed in relation to radiographic disease severity, which might have been helpful in clarifying some of the study results such as the PAM-13 scores. The patient and physician satisfaction survey collected important feedback but is not an externally validated instrument. Moreover, the study was short term, having been conducted over 90 days. Finally, the study did not include an arm in which patients used the motivating app and did not receive an injection, nor did it include an arm in which patients used the motivating app and received a saline injection (often designated as placebo).

### Practical Considerations in the Use of Mobile Health Apps

The practical aspects of incorporating a mobile health app into treatment paradigms to improve patient mobility should be further investigated. This strategy requires access to a smartphone, which may be a financial barrier for some patients [45]. Patients must also understand how the smartphone app works and have the ability and confidence to use it effectively [45]. Finally, with the ever-increasing complexity of mobile apps, continual improvements in smartphones, apps, and wearable activity monitors are needed in order to limit or prevent technical issues (and potential data loss) that may occur with routine operations such as software updates [46,47]. Continual improvement helps ensure that data are captured accurately while avoiding false positives and negatives [48,49].

Overall, the results of this study provide evidence that in patients suffering from knee OA who received hylan G-F 20, additional improvement was achieved in this study, based on mobility parameters of steps per day and pain reduction, with use of a mobile app and wearable activity monitor.

### Conclusions

Reduced mobility in patients with knee OA can be a significant issue negatively affecting quality of life and experience of pain. Increasing patients’ motivation to walk and thereby increasing their mobility may reduce these negative effects. Use of a novel smartphone app in conjunction with a wearable activity monitor provided additional improvement on mobility parameters such as steps per day and pain with walking in the 6-minute walk test in patients with knee OA who were treated with hylan G-F 20 (see Multimedia Appendix 1). These results also highlight the amenability of patients and physicians to using mobile health technology in the treatment of OA and suggest further investigation is warranted.

## Multimedia Appendix 1

Patient experience of smartphone app use during the study.

## Multimedia Appendix 2

CONSORT-EHEALTH-v1-6.

## Abbreviations

AE: adverse events
FDA: Food and Drug Administration
LS: least square
NPRS: numeric pain rating scale
OA: osteoarthritis
PAM-13: Patient Activation Measure-13
TEAE: treatment-emergent adverse events
VAMS: visual analog mood assessment
