# Supplementary material for: Assessing the Impact of a Novel Smartphone Application Compared With Standard Follow-Up on Mobility of Patients With Knee Osteoarthritis Following Treatment With Hylan G-F 20: A Randomized Controlled Trial
Source: JMIR Mhealth Uhealth. 2017 May 9;5(5):e64. doi: 10.2196/mhealth.7179 (PMC5442351; doi:10.2196/mhealth.7179)
Supplement: Supplementary file 2 [file mhealth_v5i5e64_app2.pdf]

CONSORT-EHEALTH (V 1.6.1) - Submission/Publication Form

The CONSORT-EHEALTH checklist is intended for authors of randomized trials evaluating web-based and Internet-based applications/interventions, including mobile interventions, electronic games (incl multiplayer games), social media, certain telehealth applications, and other interactive and/or networked electronic applications. Some of the items (e.g. all subitems under item 5 - description of the intervention) may also be applicable for other study designs.

The goal of the CONSORT EHEALTH checklist and guideline is to be
a) a guide for reporting for authors of RCTs,
b) to form a basis for appraisal of an ehealth trial (in terms of validity)

CONSORT-EHEALTH items/subitems are MANDATORY reporting items for studies published in the Journal of Medical Internet Research and other journals / scientific societies endorsing the checklist.

Items numbered 1., 2., 3., 4a., 4b etc are original CONSORT or CONSORT-NPT (non-pharmacologic treatment) items.
Items with Roman numerals (i., ii, iii, iv etc.) are CONSORT-EHEALTH extensions/clarifications.

As the CONSORT-EHEALTH checklist is still considered in a formative stage, we would ask that you also RATE ON A SCALE OF 1-5 how important/useful you feel each item is FOR THE PURPOSE OF THE CHECKLIST and reporting guideline (optional).

Mandatory reporting items are marked with a red *.
In the textboxes, either copy & paste the relevant sections from your manuscript into this form - please include any quotes from your manuscript in QUOTATION MARKS,
or answer directly by providing additional information not in the manuscript, or elaborating on why the item was not relevant for this study.

YOUR ANSWERS WILL BE PUBLISHED AS A SUPPLEMENTARY FILE TO YOUR PUBLICATION IN JMIR AND ARE CONSIDERED PART OF YOUR PUBLICATION (IF ACCEPTED).
Please fill in these questions diligently. Information will not be copyedited, so please use proper spelling and grammar, use correct capitalization, and avoid abbreviations.

DO NOT FORGET TO SAVE AS PDF _AND_ CLICK THE SUBMIT BUTTON SO YOUR ANSWERS ARE IN OUR DATABASE !!!

Citation Suggestion (if you append the pdf as Appendix we suggest to cite this paper in the caption):
Eysenbach G, CONSORT-EHEALTH Group
CONSORT-EHEALTH: Improving and Standardizing Evaluation Reports of Web-based and Mobile Health Interventions
J Med Internet Res 2011;13(4):e126
URL: http://www.jmir.org/2011/4/e126/
doi: 10.2196/jmir.1923
PMID: 22209829

* Required

Your name *

First Last

Nebojsa, Skrepnik

Primary Affiliation (short), City, Country *

University of Toronto, Toronto, Canada

Tucson Orthopaedic Institute, Tucson, AZ

Your e-mail address *

abc@gmail.com

NSkrepnik@tucsonortho.com

Title of your manuscript *

Provide the (draft) title of your manuscript.

Randomized study assessing the impact of a novel smartphone application compared with standard follow-up on mobility of patients with knee osteoarthritis following treatment with hylan G-F 20

Article Preparation Status/Stage *

At which stage in your article preparation are you currently (at the time you fill in this form)

- not submitted yet - in early draft status

- not submitted yet - in late draft status, just before submission
- submitted to a journal but not reviewed yet
- submitted to a journal and after receiving initial reviewer comments
- submitted to a journal and accepted, but not published yet
- published
- Other:

Revised

Journal *

If you already know where you will submit this paper (or if it is already submitted), please provide the journal name (if it is not JMIR, provide the journal name under "other")

- not submitted yet / unclear where I will submit this
- Journal of Medical Internet Research (JMIR)
- Other:

Manuscript tracking number *

If this is a JMIR submission, please provide the manuscript tracking number under "other" (The ms tracking number can be found in the submission acknowledgement email, or when you login as author in JMIR. If the paper is already published in JMIR, then the ms tracking number is the four-digit number at the end of the DOI, to be found at the bottom of each published article in JMIR)

- no ms number (yet) / not (yet) submitted to / published in JMIR
- Other: 7179

Revised

TITLE AND ABSTRACT

1a) TITLE: Identification as a randomized trial in the title

1a) Does your paper address CONSORT item 1a? *

I.e does the title contain the phrase "Randomized Controlled Trial"? (if not, explain the reason under "other")

- yes
- Other:

1a-i) Identify the mode of delivery in the title

Identify the mode of delivery. Preferably use "web-based" and/or "mobile" and/or "electronic game" in the title. Avoid ambiguous terms like "online", "virtual", "interactive". Use "Internet-based" only if Intervention includes non-web-based Internet components (e.g. email), use "computer-based" or "electronic" only if offline products are used. Use "virtual" only in the context of "virtual reality" (3-D worlds). Use "online" only in the context of "online support groups". Complement or substitute product names with broader terms for the class of products (such as "mobile" or "smart phone" instead of "iphone"), especially if the application runs on different platforms.

	1	2	3	4	5	
subitem not at all important	○	○	○	○	●	essential

Does your paper address subitem 1a-i? *

Copy and paste relevant sections from manuscript title (include quotes in quotation marks "like this" to indicate direct quotes from your manuscript), or elaborate on this item by providing additional information not in the ms, or briefly explain why the item is not applicable/relevant for your study

"impact of a novel smartphone application"

This is a required question

1a-ii) Non-web-based components or important co-interventions in title

Mention non-web-based components or important co-interventions in title, if any (e.g., "with telephone support").

	1	2	3	4	5	
subitem not at all important	○	●	○	○	○	essential

Does your paper address subitem 1a-ii?

Copy and paste relevant sections from manuscript title (include quotes in quotation marks "like this" to indicate direct quotes from your manuscript), or elaborate on this item by providing additional information not in the ms, or briefly explain why the item is not applicable/relevant for your study

"Co-interventions not applicable for this study"

1a-iii) Primary condition or target group in the title

Mention primary condition or target group in the title, if any (e.g., "for children with Type I Diabetes")
Example: A Web-based and Mobile Intervention with Telephone Support for Children with Type I Diabetes: Randomized Controlled Trial

	1	2	3	4	5	
subitem not at all important	○	○	○	○	●	essential

Does your paper address subitem 1a-iii? *

Copy and paste relevant sections from manuscript title (include quotes in quotation marks "like this" to indicate direct quotes from your manuscript), or elaborate on this item by providing additional information not in the ms, or briefly explain why the item is not applicable/relevant for your study

"patients with knee osteoarthritis"

1b) ABSTRACT: Structured summary of trial design, methods, results, and conclusions

NPT extension: Description of experimental treatment, comparator, care providers, centers, and blinding status.

1b-i) Key features/functionalities/components of the intervention and comparator in the METHODS section of the ABSTRACT

Mention key features/functionalities/components of the intervention and comparator in the abstract. If possible, also mention theories and principles used for designing the site. Keep in mind the needs of systematic reviewers and indexers by including important synonyms. (Note: Only report in the abstract what the main paper is reporting. If this information is missing from the main body of text, consider adding it)

	1	2	3	4	5	
subitem not at all important	◯	◯	◉	◯	◯	essential

Does your paper address subitem 1b-i? *

Copy and paste relevant sections from the manuscript abstract (include quotes in quotation marks "like this" to indicate direct quotes from your manuscript), or elaborate on this item by providing additional information not in the ms, or briefly explain why the item is not applicable/relevant for your study

"Patients with knee OA aged 30 -80 years who were eligible to receive hylan G-F 20 and were familiar with smartphone technology were enrolled in this randomized, multicenter, open-label study. Patients who had a body mass index (BMI) >35 were excluded. All patients received a single 6-mL injection of hylan G-F 20 and wore the Jawbone monitor. The patients were then randomized 1:1 to Jawbone and OA GO (Group A; n=107) with visible feedback ("unblinded") or Jawbone only (Group B; n=104) with no visible feedback ("blinded"). The primary endpoint was mean change from baseline in steps per day at day 90 between Groups A and B."

Revised

1b-ii) Level of human involvement in the METHODS section of the ABSTRACT

Clarify the level of human involvement in the abstract, e.g., use phrases like "fully automated" vs. "therapist/nurse/care provider/physician-assisted" (mention number and expertise of providers involved, if any). (Note: Only report in the abstract what the main paper is reporting. If this information is missing from the main body of text, consider adding it)

	1	2	3	4	5	
subitem not at all important	◯	◯	◉	◯	◯	essential

Does your paper address subitem 1b-ii?

Copy and paste relevant sections from the manuscript abstract (include quotes in quotation marks "like this" to indicate direct quotes from your manuscript), or elaborate on this item by providing additional information not in the ms, or briefly explain why the item is not applicable/relevant for your study

"All patients received a single 6-mL injection of hylan G-F 20 and wore the Jawbone monitor. The patients were then randomized 1:1 to Jawbone and OA GO (Group A; n=107) with visible feedback ("unblinded") or Jawbone only (Group B; n=104) with no visible feedback ("blinded")."

Revised

1b-iii) Open vs. closed, web-based (self-assessment) vs. face-to-face assessments in the METHODS section of the ABSTRACT

Mention how participants were recruited (online vs. offline), e.g., from an open access website or from a clinic or a closed online user group (closed usergroup trial), and clarify if this was a purely web-based trial, or there were face-to-face components (as part of the intervention or for assessment). Clearly say if outcomes were self-assessed through questionnaires (as common in web-based trials). Note: In traditional offline trials, an open trial (open-label trial) is a type of clinical trial in which both the researchers and participants know which treatment is being administered. To avoid confusion, use "blinded" or "unblinded" to indicated the level of blinding instead of "open", as "open" in web-based trials usually refers to "open access" (i.e. participants can self-enrol). (Note: Only report in the abstract what the main paper is reporting. If this information is missing from the main body of text, consider adding it)

	1	2	3	4	5	
subitem not at all important	○	○	●	○	○	essential

Does your paper address subitem 1b-iii?

Copy and paste relevant sections from the manuscript abstract (include quotes in quotation marks "like this" to indicate direct quotes from your manuscript), or elaborate on this item by providing additional information not in the ms, or briefly explain why the item is not applicable/relevant for your study

"open-label study" "The primary endpoint was mean change from baseline in steps per day at day 90 between Groups A and B."

Revised

1b-iv) RESULTS section in abstract must contain use data

Report number of participants enrolled/assessed in each group, the use/uptake of the intervention (e.g., attrition/adherence metrics, use over time, number of logins etc.), in addition to primary/secondary outcomes. (Note: Only report in the abstract what the main paper is reporting. If this information is missing from the main body of text, consider adding it)

1 2 3 4 5

subitem not at all important ○ ○ ○ ○ ● essential

Does your paper address subitem 1b-iv?

Copy and paste relevant sections from the manuscript abstract (include quotes in quotation marks "like this" to indicate direct quotes from your manuscript), or elaborate on this item by providing additional information not in the ms, or briefly explain why the item is not applicable/relevant for your study

"Baseline characteristics were similar between groups. There were significant differences between the increases in least squares (LS) mean number of steps/day (1199 vs 467, *P* = .03) and the mean percentage change (35.8% vs 11.5%, *P* = .02) from baseline in favor of Group A over Group B. There was a greater reduction in pain from baseline during the 6-minute walk test in Group A versus Group B. (LS mean change: −55.3 vs −33.8, *P* = .007). Most patients (65.4%) and surveys of physicians (67.3%) reported they would they would be likely or very likely to use/recommend the devices. Patient Activity Measure-13 scores improved from baseline (LS mean change for Groups A and B: 5.0 vs 6.9), with no significant differences between groups. The occurrence of adverse events was similar in the 2 groups."

Revised

1b-v) CONCLUSIONS/DISCUSSION in abstract for negative trials

Conclusions/Discussions in abstract for negative trials: Discuss the primary outcome - if the trial is negative (primary outcome not changed), and the intervention was not used, discuss whether negative results are attributable to lack of uptake and discuss reasons. (Note: Only report in the abstract what the main paper is reporting. If this information is missing from the main body of text, consider adding it)

1 2 3 4 5

subitem not at all important ○ ○ ○ ● ○ essential

Does your paper address subitem 1b-v?

Copy and paste relevant sections from the manuscript abstract (include quotes in quotation marks "like this" to indicate direct quotes from your manuscript), or elaborate on this item by providing additional information not in the ms, or briefly explain why the item is not applicable/relevant for your study

Not applicable, this was not a negative trial.

INTRODUCTION

2a) In INTRODUCTION: Scientific background and explanation of rationale

2a-i) Problem and the type of system/solution

Describe the problem and the type of system/solution that is object of the study: intended as stand-alone intervention vs. incorporated in broader health care program? Intended for a particular patient population? Goals of the intervention, e.g., being more cost-effective to other interventions, replace or complement other solutions? (Note: Details about the intervention are provided in “Methods” under 5)

	1	2	3	4	5	
subitem not at all important	○	○	○	○	●	essential

Does your paper address subitem 2a-i? *

Copy and paste relevant sections from the manuscript (include quotes in quotation marks "like this" to indicate direct quotes from your manuscript), or elaborate on this item by providing additional information not in the ms, or briefly explain why the item is not applicable/relevant for your study

"Walking has been shown to significantly reduce symptoms and the risk of functional limitations due to knee OA [19,20]. In one study, patients with OA of the knee who walked ≥6,000 steps/day reduced the risk of developing a functional limitation by half within the next 2 years [19]. This suggests that walking may maintain knee function. However, strategies to encourage adherence to regular walking are clearly needed. The use of mobile applications (apps) in health care may be one such strategy to increase physical activity and enhance OA management. However, there are currently no published data on the use of mobile apps for the management of knee OA."

2a-ii) Scientific background, rationale: What is known about the (type of) system

Scientific background, rationale: What is known about the (type of) system that is the object of the study (be sure to discuss the use of similar systems for other conditions/diagnoses, if appropiate), motivation for the study, i.e. what are the reasons for and what is the context for this specific study, from which stakeholder viewpoint is the study performed, potential impact of findings [2]. Briefly justify the choice of the comparator.

	1	2	3	4	5	
subitem not at all important	○	○	●	○	○	essential

Does your paper address subitem 2a-ii? *

Copy and paste relevant sections from the manuscript (include quotes in quotation marks "like this" to indicate direct quotes from your manuscript), or elaborate on this item by providing additional information not in the ms, or briefly explain why the item is not applicable/relevant for your study

"To date, there is no disease-modifying therapy available for OA [11]. Optimal management of knee OA requires a combination of both pharmacologic (eg, oral or topical analgesics or intra-articular therapies [viscosupplementation, corticosteroids]) and nonpharmacologic methods (eg, weight loss, exercise, and physical activity) [12-15]. However, despite the well-recognized benefits of exercise and the promotion of physical activity by many professional societies [12,13,15], there is no standard exercise or education program and no clear benefit of one exercise program over another [13]. Information about the benefits of exercise is readily available [12,13,15] but rarely incorporated into patient behavior [16,17], and adherence to such programs by patients with knee OA is poor [16,17]. There is also no consensus on which measures or combination of measures should be used to assess physical function in patients with knee OA [18], complicating the initial assessment and tracking of progress in mobility improvement."

2b) In INTRODUCTION: Specific objectives or hypotheses

Does your paper address CONSORT subitem 2b? *

Copy and paste relevant sections from the manuscript (include quotes in quotation marks "like this" to indicate direct quotes from your manuscript), or elaborate on this item by providing additional information not in the ms, or briefly explain why the item is not applicable/relevant for your study

"The objective of the current study was to examine the impact on mobility in knee OA patients who were treated with hylan G-F 20 and standard of care follow up plus a mobile smartphone app (OA GO) and a wearable activity monitor (Jawbone® UP 24™) versus hylan G-F 20 and standard of care follow up alone over 90 days. We hypothesized that patients using the OA GO app and a wearable activity monitor would experience a positive impact on their mobility compared with those not using the app."

Revised

METHODS

3a) Description of trial design (such as parallel, factorial) including allocation ratio

Does your paper address CONSORT subitem 3a? *

Copy and paste relevant sections from the manuscript (include quotes in quotation marks "like this" to indicate direct quotes from your manuscript), or elaborate on this item by providing additional information not in the ms, or briefly explain why the item is not applicable/relevant for your study

"Consecutive patients with knee OA whom the physician investigator decided to treat with one 6-mL injection of hylan G-F 20 (in accordance with the US label) who consented and qualified were enrolled in this prospective, open-label, multicenter, randomized, parallel-group study.""Patients were randomized 1:1 (stratified by site) to Group A or Group B."

Revised

3b) Important changes to methods after trial commencement (such as eligibility criteria), with reasons

Does your paper address CONSORT subitem 3b? *

Copy and paste relevant sections from the manuscript (include quotes in quotation marks "like this" to indicate direct quotes from your manuscript), or elaborate on this item by providing additional information not in the ms, or briefly explain why the item is not applicable/relevant for your study

No important changes to trial after commencement.

3b-i) Bug fixes, Downtimes, Content Changes

Bug fixes, Downtimes, Content Changes: ehealth systems are often dynamic systems. A description of changes to methods therefore also includes important changes made on the intervention or comparator during the trial (e.g., major bug fixes or changes in the functionality or content) (5-iii) and other "unexpected events" that may have influenced study design such as staff changes, system failures/downtimes, etc. [2].

	1	2	3	4	5	
subitem not at all important	○	○	●	○	○	essential

Does your paper address subitem 3b-i?

Copy and paste relevant sections from the manuscript (include quotes in quotation marks "like this" to indicate direct quotes from your manuscript), or elaborate on this item by providing additional information not in the ms, or briefly explain why the item is not applicable/relevant for your study

No important changes made during the trial.

4a) Eligibility criteria for participants

Does your paper address CONSORT subitem 4a? *

Revised

"Consecutive patients with knee OA whom the physician investigator decided to treat with one 6-mL injection of hylan G-F 20 (in accordance with the US label) who consented and qualified were enrolled in this prospective, open-label, multicenter, randomized, parallel-group study. Study sites and physicians were recruited using a detailed questionnaire and site qualification visit. Patients were recruited from the selected private community based practices and research only practice sites. All eligible patients were able to read and understand English and provided informed consent prior to starting the study. " "To be included in the study, all patients must have had unilateral knee OA and have been suitable for treatment with hylan G-F 20 based on the decision of the physician investigator. Study sites were requested to report whether or not a knee examination was performed or X-ray was taken but the results of these examinations were not captured." "Patients were excluded if they were aged <30 years or >80 years, were unfamiliar with smartphones, or had baseline pain >9 on the 11-point numeric pain rating scale (NPRS; pain ratings could range from 0, no pain; to 5, moderate pain; to 10, worst possible pain) in the target-for-treatment knee while walking on a flat surface. Patients with bilateral disease were excluded, with the exception of patients who were treated in only one knee and had contralateral knee pain <4 on NPRS while walking on a flat surface. Patients whose baseline daily step average was <500 or >8000 as assessed during the screening and run-in phases were not eligible. Also excluded were patients who had a body mass index (BMI) >35 or life expectancy <12 months; were currently using a wearable activity monitor or analogous device; had planned surgery on any lower extremity joint or any significant medical condition that would interfere with study participation; were chronic narcotic users; or were pregnant/breastfeeding or likely to become pregnant. The protocol did not specifically exclude patients based on use of previous intra-articular injections (cortisone or hyaluronic acid)."

	1	2	3	4	5	
subitem not at all important	○	○	○	○	●	essential

Does your paper address subitem 4a-i?

Copy and paste relevant sections from the manuscript (include quotes in quotation marks "like this" to indicate direct quotes from your manuscript), or elaborate on this item by providing additional information not in the ms, or briefly explain why the item is not applicable/relevant for your study

"Patients were excluded if they were aged <30 years or >80 years, were unfamiliar with smartphones,.."

4a-ii) Open vs. closed, web-based vs. face-to-face assessments:

Open vs. closed, web-based vs. face-to-face assessments: Mention how participants were recruited (online vs. offline), e.g., from an open access website or from a clinic, and clarify if this was a purely web-based trial, or there were face-to-face components (as part of the intervention or for assessment), i.e., to what degree got the study team to know the participant. In online-only trials, clarify if participants were quasi-anonymous and whether having multiple identities was possible or whether technical or logistical measures (e.g., cookies, email confirmation, phone calls) were used to detect/prevent these.

	1	2	3	4	5	
subitem not at all important	○	○	●	○	○	essential

Does your paper address subitem 4a-ii? *

Copy and paste relevant sections from the manuscript (include quotes in quotation marks "like this" to indicate direct quotes from your manuscript), or elaborate on this item by providing additional information not in the ms, or briefly explain why the item is not applicable/relevant for your study

Revised

"Consecutive patients with knee OA whom the physician investigator decided to treat with one 6-mL injection of hylan G-F 20 (in accordance with the US label) who consented and qualified were enrolled in this prospective, open-label, multicenter, randomized, parallel-group study. Study sites and physicians were recruited using a detailed questionnaire and site qualification visit. Patients were recruited from the selected private community based practices and research only practice sites." "All eligible patients received hylan G-F 20. Participants wore a commercially available activity monitor (Jawbone UP 24) on their wrists and were instructed to remove the monitor only during the weekly charging times and in situations where the device would be submerged in water. Patients were randomized 1:1 (stratified by site) to Group A or Group B." "Group A patients were provided with regular follow-up plus an unblinded wearable activity monitor and a mobile app (OA GO) (Figure 1). The OA GO app (downloaded to a trial-sponsored iPhone 5 or newer) provided motivational messages and requested that the patient enter pain and mood data on a once-daily basis. Trial coordinators demonstrated app use, provided charging instructions for the Jawbone UP 24, and set the daily step goal based on patient's baseline steps per day during screening."

informed consent procedures (e.g., publish the informed consent documentation as appendix, see also item X26), as this information may have an effect on user self-selection, user expectation and may also bias results.

	1	2	3	4	5	
subitem not at all important	○	○	●	○	○	essential

Does your paper address subitem 4a-iii?

Copy and paste relevant sections from the manuscript (include quotes in quotation marks "like this" to indicate direct quotes from your manuscript), or elaborate on this item by providing additional information not in the ms, or briefly explain why the item is not applicable/relevant for your study

""All eligible patients were able to read and understand English and provided informed consent prior to starting the study."

4b) Settings and locations where the data were collected

Does your paper address CONSORT subitem 4b? *

Copy and paste relevant sections from the manuscript (include quotes in quotation marks "like this" to indicate direct quotes from your manuscript), or elaborate on this item by providing additional information not in the ms, or briefly explain why the item is not applicable/relevant for your study

"Consecutive patients with knee OA whom the physician investigator decided to treat with one 6-mL injection of hylan G-F 20 (in accordance with the US label) who consented and qualified were enrolled in this prospective, open-label, multicenter, randomized, parallel-group study." "Patients were recruited from the selected private community based practices and research only practice sites." "The study consisted of 5 visits: screening and baseline (days −7 and 1) with follow-up visits at days 7, 30 and day 90, last visit. The main study duration was 90 days. A post-study adherence check in Group A patients occurred at day 180, when data were downloaded from the app (no visit)."

Revised

[illegible]n in web-based trials) or otherwise.

	1	2	3	4	5	
subitem not at all important	○	○	●	○	○	essential

Does your paper address subitem 4b-i? *

Copy and paste relevant sections from the manuscript (include quotes in quotation marks "like this" to indicate direct quotes from your manuscript), or elaborate on this item by providing additional information not in the ms, or briefly explain why the item is not applicable/relevant for your study

Outcomes were not assessed through online questionnaires.

4b-ii) Report how institutional affiliations are displayed

Report how institutional affiliations are displayed to potential participants [on ehealth media], as affiliations with prestigious hospitals or universities may affect volunteer rates, use, and reactions with regards to an intervention.(Not a required item – describe only if this may bias results)

	1	2	3	4	5	
subitem not at all important	○	○	●	○	○	essential

Does your paper address subitem 4b-ii?

Copy and paste relevant sections from the manuscript (include quotes in quotation marks "like this" to indicate direct quotes from your manuscript), or elaborate on this item by providing additional information not in the ms, or briefly explain why the item is not applicable/relevant for your study

Not applicable, institutions not displayed during this study.

5) The interventions for each group with sufficient details to allow replication, including how and when they were actually administered

5-i) Mention names, credential, affiliations of the developers, sponsors, and owners

Mention names, credential, affiliations of the developers, sponsors, and owners [6] (if authors/evaluators are owners or developer of the software, this needs to be declared in a "Conflict of interest" section or mentioned elsewhere in the manuscript).

	1	2	3	4	5	
subitem not at all important	○	○	●	○	○	essential

Does your paper address subitem 5-i?

Copy and paste relevant sections from the manuscript (include quotes in quotation marks "like this" to indicate direct quotes from your manuscript), or elaborate on this item by providing additional information not in the ms, or briefly explain why the item is not applicable/relevant for your study

"Participants wore a commercially available activity monitor (Jawbone UP 24) on their wrists and were instructed to remove the monitor only during the weekly charging times and in situations where the device would be submerged in water." The mobile app, OA GO, was developed by the sponsor of the study, Sanofi.

Revised

5-ii) Describe the history/development process

Describe the history/development process of the application and previous formative evaluations (e.g., focus groups, usability testing), as these will have an impact on adoption/use rates and help with interpreting results.

	1	2	3	4	5	
subitem not at all important	○	○	●	○	○	essential

Does your paper address subitem 5-ii?

Copy and paste relevant sections from the manuscript (include quotes in quotation marks "like this" to indicate direct quotes from your manuscript), or elaborate on this item by providing additional information not in the ms, or briefly explain why the item is not applicable/relevant for your study

Study was focused on clinical impact of using a wearable activity monitor and smartphone application and not on development of the application.

5-iii) Revisions and updating

Revisions and updating. Clearly mention the date and/or version number of the application/intervention (and comparator, if applicable) evaluated, or describe whether the intervention underwent major changes during the evaluation process, or whether the development and/or content was "frozen" during the trial. Describe dynamic components such as news feeds or changing content which may have an impact on the replicability of the intervention (for unexpected events see item 3b).

	1	2	3	4	5	
subitem not at all important	○	○	●	○	○	essential

Does your paper address subitem 5-iii?

Copy and paste relevant sections from the manuscript (include quotes in quotation marks "like this" to indicate direct quotes from your manuscript), or elaborate on this item by providing additional information not in the ms, or briefly explain why the item is not applicable/relevant for your study

Not applicable to this study. Intervention did not undergo major changes during the study. App was self-contained and not linked to external sites.

5-iv) Quality assurance methods

Provide information on quality assurance methods to ensure accuracy and quality of information provided [1], if applicable.

	1	2	3	4	5	
subitem not at all important	○	○	●	○	○	essential

Does your paper address subitem 5-iv?

Copy and paste relevant sections from the manuscript (include quotes in quotation marks "like this" to indicate direct quotes from your manuscript), or elaborate on this item by providing additional information not in the ms, or briefly explain why the item is not applicable/relevant for your study

Not applicable, app was self-contained.

5-v) Ensure replicability by publishing the source code, and/or providing screenshots/screen-capture video, and/or providing flowcharts of the algorithms used

Ensure replicability by publishing the source code, and/or providing screenshots/screen-capture video, and/or providing flowcharts of the algorithms used. Replicability (i.e., other researchers should in principle be able to replicate the study) is a hallmark of scientific reporting.

	1	2	3	4	5	
subitem not at all important	○	○	●	○	○	essential

Does your paper address subitem 5-v?

Copy and paste relevant sections from the manuscript (include quotes in quotation marks "like this" to indicate direct quotes from your manuscript), or elaborate on this item by providing additional information not in the ms, or briefly explain why the item is not applicable/relevant for your study

Screenshots of app are included in the manuscript.

5-vi) Digital preservation

Digital preservation: Provide the URL of the application, but as the intervention is likely to change or disappear over the course of the years; also make sure the intervention is archived (Internet Archive, webcitation.org, and/or publishing the source code or screenshots/videos alongside the article). As pages behind login screens cannot be archived, consider creating demo pages which are accessible without login.

1 2 3 4 5

subitem not at all important ○ ○ ● ○ ○ essential

Does your paper address subitem 5-vi?

Copy and paste relevant sections from the manuscript (include quotes in quotation marks "like this" to indicate direct quotes from your manuscript), or elaborate on this item by providing additional information not in the ms, or briefly explain why the item is not applicable/relevant for your study

Not applicable, application is not Web-based.

5-vii) Access

Access: Describe how participants accessed the application, in what setting/context, if they had to pay (or were paid) or not, whether they had to be a member of specific group. If known, describe how participants obtained "access to the platform and Internet" [1]. To ensure access for editors/reviewers/readers, consider to provide a "backdoor" login account or demo mode for reviewers/readers to explore the application (also important for archiving purposes, see vi).

1 2 3 4 5

subitem not at all important ○ ○ ● ○ ○ essential

Does your paper address subitem 5-vii? *

Copy and paste relevant sections from the manuscript (include quotes in quotation marks "like this" to indicate direct quotes from your manuscript), or elaborate on this item by providing additional information not in the ms, or briefly explain why the item is not applicable/relevant for your study

"Patients were randomized 1:1 (stratified by site) to Group A or Group B. Group A patients were provided with regular follow-up plus an unblinded wearable activity monitor and a mobile app (OA GO) (Figure 1). The OA GO app (downloaded to a trial-sponsored iPhone 5 or newer) provided motivational messages and requested that the patient enter pain and mood data on a once-daily basis." "Group B was provided with regular follow-up plus a blinded wearable activity monitor, with instructions to wear the monitor at all times except during weekly charging and water activities. The patients in Group B received standard-of-care instructions and education (information on the benefits of walking in a brochure available from the Arthritis Foundation) but did not have access to activity recorded by the wearable activity monitor."

5-viii) Mode of delivery, features/functionalities/components of the intervention and comparator, and the theoretical framework

Describe mode of delivery, features/functionalities/components of the intervention and comparator, and the theoretical framework [6] used to design them (instructional strategy [1], behaviour change techniques, persuasive features, etc., see e.g., [7, 8] for terminology). This includes an in-depth description of the content (including where it is coming from and who developed it) [1]," whether [and how] it is tailored to individual circumstances and allows users to track their progress and receive feedback" [6]. This also includes a description of communication delivery channels and – if computer-mediated communication is a component – whether communication was synchronous or asynchronous [6]. It also includes information on presentation strategies [1], including page design principles, average amount of text on pages, presence of hyperlinks to other resources, etc. [1].

	1	2	3	4	5	
subitem not at all important	○	○	●	○	○	essential

Does your paper address subitem 5-viii? *

Copy and paste relevant sections from the manuscript (include quotes in quotation marks "like this" to indicate direct quotes from your manuscript), or elaborate on this item by providing additional information not in the ms, or briefly explain why the item is not applicable/relevant for your study

"The OA GO app (downloaded to a trial-sponsored iPhone 5 or newer) provided motivational messages and requested that the patient enter pain and mood data on a once-daily basis." "The OA GO app combined continuously retrieved data from the wearable activity monitor with data entered by the patient to display the patient's daily step count, calories burned, and sleep. Daily and monthly cumulative activity trends were available for the patient to review."

5-ix) Describe use parameters

Describe use parameters (e.g., intended "doses" and optimal timing for use). Clarify what instructions or recommendations were given to the user, e.g., regarding timing, frequency, heaviness of use, if any, or was the intervention used ad libitum.

	1	2	3	4	5	
subitem not at all important	○	○	●	○	○	essential

Does your paper address subitem 5-ix?

Copy and paste relevant sections from the manuscript (include quotes in quotation marks "like this" to indicate direct quotes from your manuscript), or elaborate on this item by providing additional information not in the ms, or briefly explain why the item is not applicable/relevant for your study

"The OA GO app (downloaded to a trial-sponsored iPhone 5 or newer) provided motivational messages and requested that the patient enter pain and mood data on a once-daily basis. Trial coordinators demonstrated app use, provided charging instructions for the Jawbone UP 24, and set the daily step goal based on patient's baseline steps per day during screening. The OA GO app combined continuously retrieved data from the wearable activity monitor with data entered by the patient to display the patient's daily step count, calories burned, and sleep. Daily and monthly cumulative activity trends were available for the patient to review."

5-x) Clarify the level of human involvement

Clarify the level of human involvement (care providers or health professionals, also technical assistance) in the e-intervention or as co-intervention (detail number and expertise of professionals involved, if any, as well as "type of assistance offered, the timing and frequency of the support, how it is initiated, and the medium by which the assistance is delivered". It may be necessary to distinguish between the level of human involvement required for the trial, and the level of human involvement required for a routine application outside of a RCT setting (discuss under item 21 – generalizability).

	1	2	3	4	5	
subitem not at all important	○	○	●	○	○	essential

Does your paper address subitem 5-x?

Copy and paste relevant sections from the manuscript (include quotes in quotation marks "like this" to indicate direct quotes from your manuscript), or elaborate on this item by providing additional information not in the ms, or briefly explain why the item is not applicable/relevant for your study

Group A "Trial coordinators demonstrated app use, provided charging instructions for the Jawbone UP 24, and set the daily step goal based on patient's baseline steps per day during screening." "The patients in Group B received standard-of-care instructions and education (information on the benefits of walking in a brochure available from the Arthritis Foundation) but did not have access to activity recorded by the wearable activity monitor." "The study consisted of 5 visits: screening and baseline (days −7 and 1) with follow-up visits at days 7, 30 and day 90, last visit."

Revised

5-xi) Report any prompts/reminders used

Report any prompts/reminders used: Clarify if there were prompts (letters, emails, phone calls, SMS) to use the application, what triggered them, frequency etc. It may be necessary to distinguish between the level of prompts/reminders required for the trial, and the level of prompts/reminders for a routine application outside of a RCT setting (discuss under item 21 – generalizability).

	1	2	3	4	5	
subitem not at all important	○	○	●	○	○	essential

Does your paper address subitem 5-xi? *

Copy and paste relevant sections from the manuscript (include quotes in quotation marks "like this" to indicate direct quotes from your manuscript), or elaborate on this item by providing additional information not in the ms, or briefly explain why the item is not applicable/relevant for your study

Investigators did not provide prompts to use the app.

5-xii) Describe any co-interventions (incl. training/support)

Describe any co-interventions (incl. training/support): Clearly state any interventions that are provided in addition to the targeted eHealth intervention, as ehealth intervention may not be designed as stand-alone intervention. This includes training sessions and support [1]. It may be necessary to distinguish between the level of training required for the trial, and the level of training for a routine application outside of a RCT setting (discuss under item 21 – generalizability.

	1	2	3	4	5	
subitem not at all important	○	○	●	○	○	essential

Does your paper address subitem 5-xii? *

Copy and paste relevant sections from the manuscript (include quotes in quotation marks "like this" to indicate direct quotes from your manuscript), or elaborate on this item by providing additional information not in the ms, or briefly explain why the item is not applicable/relevant for your study

"Trial coordinators demonstrated app use, provided charging instructions for the Jawbone UP 24, and set the daily step goal based on patient's baseline steps per day during screening."

6a) Completely defined pre-specified primary and secondary outcome measures, including how and when they were assessed

"The primary endpoint was mean change from baseline to day 90 in mobility as measured by steps per day. Baseline steps per day was the average over at least 3 days, and last assessment was the average steps per day over the 7 days immediately preceding the last visit. Change was calculated as the difference between the 2 averages. Use of average steps per day avoided bias due to any one particularly good or bad day for the patient. The secondary endpoints were mean percentage change from baseline in steps per day at each assessment visit (average of a 7-day period); and at day 90, mean percentage change from baseline in the 6-minute walk test (distance and pain assessed by the NPRS); patient and physician satisfaction with treatment; percentage change in Patient Activation Measure (PAM)-13 questionnaire score [21]; percentage change in sleep captured by the wearable activity monitor (light, sound, and duration of sleep); and Visual Analog Mood Scale VAMS) assessment. Treatment-emergent adverse events (TEAEs) were also assessed.
For Group A, the satisfaction of patients and physicians was captured using survey questionnaires. The patient survey consisted of 7 questions: the first 6 were answered on a 7-point Likert scale related to the extent to which the patients experienced changes (−3 to +3, where 0=no change), and the seventh was a 4-point scale asking about their likelihood of using the device in the future (not at all likely to very likely). Valid questionnaires were those in which at least 4 of the first 6 questions were answered. A physician survey was similarly constructed with a final question, using the 4-point scale, which asked whether they would recommend the device in the future (not likely to very likely). Physicians answered the survey at day 90, before viewing the data, and must have answered at least 3 of the first 4 questions. All responses to survey questions were based on patient and physician observations and opinions."
The PAM-13 [21] was used to assess patients' knowledge, skills, and confidence for self-management at randomization and last visit, with responses given on a Guttman-like scale that ranged from 1 to 4 (strongly disagree to strongly agree). Unanswered items were scored as missing. Raw scores ranged from 13 to 52, with higher scores indicating more activation; raw scores were converted to activation scores, which ranged from 0 to 100, with a score of 100 corresponding to the highest degree of activation."

	1	2	3	4	5	
subitem not at all important	○	○	○	○	●	essential

Does your paper address subitem 6a-i?

Copy and paste relevant sections from manuscript text

Not applicable. The study did not use online questionnaires.

6a-ii) Describe whether and how "use" (including intensity of use/dosage) was defined/measured/monitored

Describe whether and how "use" (including intensity of use/dosage) was defined/measured/monitored (logins, logfile analysis, etc.). Use/adoption metrics are important process outcomes that should be reported in any ehealth trial.

1 2 3 4 5

subitem not at all important ○ ○ ● ○ ○ essential

Does your paper address subitem 6a-ii?

Copy and paste relevant sections from manuscript text

"A post-study adherence check in Group A patients occurred at day 180, when data were downloaded from the app (no visit)."

6a-iii) Describe whether, how, and when qualitative feedback from participants was obtained

Describe whether, how, and when qualitative feedback from participants was obtained (e.g., through emails, feedback forms, interviews, focus groups).

1 2 3 4 5

subitem not at all important ○ ○ ● ○ ○ essential

Does your paper address subitem 6a-iii?

Copy and paste relevant sections from manuscript text

"For Group A, the satisfaction of patients and physicians was captured using survey questionnaires. The patient survey consisted of 7 questions: the first 6 were answered on a 7-point Likert scale related to the extent to which the patients experienced changes (−3 to +3, where 0=no change), and the seventh was a 4-point scale asking about their likelihood of using the device in the future (not at all likely to very likely). Valid questionnaires were those in which at least 4 of the first 6 questions were answered. A physician survey was similarly constructed with a final question, using the 4-point scale, which asked whether they would recommend the device in the future (not likely to very likely). Physicians answered the survey at day 90, before viewing the data, and must have answered at least 3 of the first 4 questions. All responses to survey questions were based on patient and physician observations and opinions."

6b) Any changes to trial outcomes after the trial commenced, with reasons

Does your paper address CONSORT subitem 6b? *

Copy and paste relevant sections from the manuscript (include quotes in quotation marks "like this" to indicate direct quotes from your manuscript), or elaborate on this item by providing additional information not in the ms, or briefly explain why the item is not applicable/relevant for your study

No changes to trial outcomes after the trial commenced.

7a) How sample size was determined

NPT: When applicable, details of whether and how the clustering by care provides or centers was addressed

7a-i) Describe whether and how expected attrition was taken into account when calculating the sample size

Describe whether and how expected attrition was taken into account when calculating the sample size.

	1	2	3	4	5	
subitem not at all important	○	○	●	○	○	essential

Does your paper address subitem 7a-i?

Copy and paste relevant sections from manuscript title (include quotes in quotation marks "like this" to indicate direct quotes from your manuscript), or elaborate on this item by providing additional information not in the ms, or briefly explain why the item is not applicable/relevant for your study

"A sample size of 200 randomized (172 evaluable) patients (100 randomized [86 evaluable] per group) was estimated to provide 80% power to detect an average increase in the change from baseline steps per day of 25% for Group A compared with Group B, assuming a 2-sided significance level of 5%. The variability was expected to be similar in the 2 groups, and an estimate of 58% for the coefficient of variation was used in this calculation [24]. The sample size of 200 randomized patients assumed a drop-out rate of 15%"

7b) When applicable, explanation of any interim analyses and stopping guidelines

Does your paper address CONSORT subitem 7b? *

Copy and paste relevant sections from the manuscript (include quotes in quotation marks "like this" to indicate direct quotes from your manuscript), or elaborate on this item by providing additional information not in the ms, or briefly explain why the item is not applicable/relevant for your study

Not relevant for this study, there were no interim analyses.

8a) Method used to generate the random allocation sequence

NPT: When applicable, how care providers were allocated to each trial group

Does your paper address CONSORT subitem 8a? *

Copy and paste relevant sections from the manuscript (include quotes in quotation marks "like this" to indicate direct quotes from your manuscript), or elaborate on this item by providing additional information not in the ms, or briefly explain why the item is not applicable/relevant for your study

"The randomization scheme was generated by the study sponsor and stratified by site. Sealed envelopes, numbered in an ascending order for use, were provided to each site. The envelopes were opened according to ascending sequence to ensure proper randomization."

Revised

8b) Type of randomisation; details of any restriction (such as blocking and block size)

Does your paper address CONSORT subitem 8b? *

Copy and paste relevant sections from the manuscript (include quotes in quotation marks "like this" to indicate direct quotes from your manuscript), or elaborate on this item by providing additional information not in the ms, or briefly explain why the item is not applicable/relevant for your study

"Patients were randomized 1:1 (stratified by site) to Group A or Group B."

9) Mechanism used to implement the random allocation sequence (such as sequentially numbered containers), describing any steps taken to conceal the sequence until interventions were assigned

Does your paper address CONSORT subitem 9? *

Copy and paste relevant sections from the manuscript (include quotes in quotation marks "like this" to indicate direct quotes from your manuscript), or elaborate on this item by providing additional information not in the ms, or briefly explain why the item is not applicable/relevant for your study

Randomization envelopes containing a six-digit randomization number were used. The envelope contained places for the signature of the person opening the envelope, the nine digit patient identification number, and the date and time the envelope was opened. After entering the patient number, date and time, the envelope could be opened where the site would find a card containing the same randomization number that appeared on the envelope and the treatment assignment. The envelope with its contents was put in the patient's file.

10) Who generated the random allocation sequence, who enrolled participants, and who assigned participants to interventions

Does your paper address CONSORT subitem 10? *

Copy and paste relevant sections from the manuscript (include quotes in quotation marks "like this" to indicate direct quotes from your manuscript), or elaborate on this item by providing additional information not in the ms, or briefly explain why the item is not applicable/relevant for your study

"Consecutive patients with knee OA whom the physician investigator decided to treat with one 6-mL injection of hylan G-F 20 (in accordance with the US label) who consented and qualified were enrolled in this open-label, multicenter, randomized, parallel-group study.""Patients were randomized 1:1 (stratified by site) to Group A or Group B. The randomization scheme was generated by the study sponsor and stratified by site."

Revised

11a) If done, who was blinded after assignment to interventions (for example, participants, care providers, those assessing outcomes) and how

NPT: Whether or not administering co-interventions were blinded to group assignment

11a-i) Specify who was blinded, and who wasn't

Specify who was blinded, and who wasn't. Usually, in web-based trials it is not possible to blind the participants [1, 3] (this should be clearly acknowledged), but it may be possible to blind outcome assessors, those doing data analysis or those administering co-interventions (if any).

1 2 3 4 5

subitem not at all important ○ ○ ● ○ ○ essential

Does your paper address subitem 11a-i? *

Copy and paste relevant sections from the manuscript (include quotes in quotation marks "like this" to indicate direct quotes from your manuscript), or elaborate on this item by providing additional information not in the ms, or briefly explain why the item is not applicable/relevant for your study

Not applicable. This was an open-label study of consecutive patients treated by investigators.

Revised

11a-ii) Discuss e.g., whether participants knew which intervention was the "intervention of interest" and which one was the "comparator"

Informed consent procedures (4a-ii) can create biases and certain expectations - discuss e.g., whether participants knew which intervention was the "intervention of interest" and which one was the "comparator".

1 2 3 4 5

subitem not at all important ○ ● ○ ○ ○ essential

Does your paper address subitem 11a-ii?

Copy and paste relevant sections from the manuscript (include quotes in quotation marks "like this" to indicate direct quotes from your manuscript), or elaborate on this item by providing additional information not in the ms, or briefly explain why the item is not applicable/relevant for your study

Not applicable, this was an open-label study.

Revised

11b) If relevant, description of the similarity of interventions

(this item is usually not relevant for ehealth trials as it refers to similarity of a placebo or sham intervention to a active medication/intervention)

Does your paper address CONSORT subitem 11b? *

Copy and paste relevant sections from the manuscript (include quotes in quotation marks "like this" to indicate direct quotes from your manuscript), or elaborate on this item by providing additional information not in the ms, or briefly explain why the item is not applicable/relevant for your study

"Participants wore a commercially available activity monitor (Jawbone UP 24) on their wrists and were instructed to remove the monitor only during the weekly charging times and in situations where the device would be submerged in water." Both groups received "...standard-of-care instructions (information on the benefits of walking in a brochure available from the Arthritis Foundation)..."

Revised

12a) Statistical methods used to compare groups for primary and secondary outcomes

NPT: When applicable, details of whether and how the clustering by care providers or centers was addressed

Does your paper address CONSORT subitem 12a? *

Copy and paste relevant sections from the manuscript (include quotes in quotation marks "like this" to indicate direct quotes from your manuscript), or elaborate on this item by providing a[illegible] not in the ms, or briefly explain why the item is not applicable/relevant for your study

Revised

"All efficacy endpoints were analyzed in the modified intent-to-treat population, which included all randomized patients who had both a baseline value and an on-treatment value for the primary endpoint on or after day 30. All efficacy endpoint data comparing change from baseline between groups were obtained from an analysis of covariance model with baseline mean steps per day as the covariate and treatment assignment and pooled site as class variables. They are presented as least squares (LS) means with 95% confidence intervals (CIs). Statistical analyses were based on initial verification of parametric model assumptions, and a rank analysis of covariance was then considered if conditions for a parametric model were not met."

12a-i) Imputation techniques to deal with attrition / missing values

Imputation techniques to deal with attrition / missing values: Not all participants will use the intervention/comparator as intended and attrition is typically high in ehealth trials. Specify how participants who did not use the application or dropped out from the trial were treated in the statistical analysis (a complete case analysis is strongly discouraged, and simple imputation techniques such as LOCF may also be problematic [4]).

	1	2	3	4	5	
subitem not at all important	○	○	●	○	○	essential

Does your paper address subitem 12a-i? *

Copy and paste relevant sections from the manuscript (include quotes in quotation marks "like this" to indicate direct quotes from your manuscript), or elabora[illegible]ding additional information not in the ms, or briefly explain why the item is not applic[illegible]study

Revised

"All efficacy endpoints were analyzed in the modified intent-to-treat population, which included all randomized patients who had both a baseline value and an on-treatment value for the primary endpoint on or after day 30." In cases of permanent discontinuation of device use, the patient data from the last visit were considered as last assessment in data analysis.

12b) Methods for additional analyses, such as subgroup analyses and adjusted analyses

Does your paper address CONSORT subitem 12b? *

Copy and paste relevant sections from the manuscript (include quotes in quotation marks "like this" to indicate direct quotes from your manuscript), or elaborate on this item by providing additional information not in the ms, or briefly explain why the item is not applicable/relevant for your study

Primary and secondary endpoints were analyzed and presented in this manuscript.

X26) REB/IRB Approval and Ethical Considerations [recommended as subheading under "Methods"] (not a CONSORT item)

X26-i) Comment on ethics committee approval

	1	2	3	4	5	
subitem not at all important	○	○	○	○	●	essential

Does your paper address subitem X26-i?

Copy and paste relevant sections from the manuscript (include quotes in quotation marks "like this" to indicate direct quotes from your manuscript), or elaborate on this item by providing additional information not in the ms, or briefly explain why the item is not applicable/relevant for your study

"The protocol complied with the 18th World Health Congress (Helsinki, 1964) recommendations and applicable amendments, and with any applicable country-specific laws, regulations, and guidelines. The protocol was approved by relevant ethics committees and/or institutional review boards."

x26-ii) Outline informed consent procedures

Outline informed consent procedures e.g., if consent was obtained offline or online (how? Checkbox, etc.?), and what information was provided (see 4a-ii). See [6] for some items to be included in informed consent documents.

	1	2	3	4	5	
subitem not at all important	○	○	○	○	●	essential

Does your paper address subitem X26-ii?

Copy and paste relevant sections from the manuscript (include quotes in quotation marks "like this" to indicate direct quotes from your manuscript), or elaborate on this item by providing additional information not in the ms, or briefly explain why the item is not applicable/relevant for your study

The patients must have been able to read, understand and sign an informed consent form, understand requirements for follow-up visits and must have been willing to provide information at the scheduled evaluations.

X26-iii) Safety and security procedures

Safety and security procedures, incl. privacy considerations, and any steps taken to reduce the likelihood or detection of harm (e.g., education and training, availability of a hotline)

	1	2	3	4	5	
subitem not at all important	○	○	○	●	○	essential

Does your paper address subitem X26-iii?

Copy and paste relevant sections from the manuscript (include quotes in quotation marks "like this" to indicate direct quotes from your manuscript), or elaborate on this item by providing additional information not in the ms, or briefly explain why the item is not applicable/relevant for your study

Temporary discontinuation may have been considered by the investigator because of suspected adverse events or complaint. The patients may have withdrawn from treatment if they decided to do so, at any time and irrespective of the reason, or this may have been the investigator's decision.

RESULTS

13a) For each group, the numbers of participants who were randomly assigned, received intended treatment, and were analysed for the primary outcome

NPT: The number of care providers or centers performing the intervention in each group and the number of patients treated by each care provider in each center

Does your paper address CONSORT subitem 13a? *

Copy and paste relevant sections from the manuscript (include quotes in quotation marks "like this" to indicate direct quotes from your manuscript), or elaborate on this item by providing additional information not in the ms, or briefly explain why the item is not applicable/relevant for your study

"A total of 211 knee OA patients treated with hylan G-F 20 were randomized (Group A=107; Group B=104), and nearly all patients completed the 90-day observation period (Group A, 104 [97.2%]; Group B, 103 [99%]) (Figure 2)."

13b) For each group, losses and exclusions after randomisation, together with reasons

Does your paper address CONSORT subitem 13b? (NOTE: Preferably, this is shown in a CONSORT flow diagram) *

Copy and paste relevant sections from the manuscript (include quotes in quotation marks "like this" to indicate direct quotes from your manuscript), or elaborate on this item by providing additional information not in the ms, or briefly explain why the item is not applicable/relevant for your study

Please see Figure 2 in the manuscript for this information.

13b-i) Attrition diagram

Strongly recommended: An attrition diagram (e.g., proportion of participants still logging in or using the intervention/comparator in each group plotted over time, similar to a survival curve) or other figures or tables demonstrating usage/dose/engagement.

	1	2	3	4	5	
subitem not at all important	○	○	●	○	○	essential

Does your paper address subitem 13b-i?

Copy and paste relevant sections from the manuscript or cite the figure number if applicable (include quotes in quotation marks "like this" to indicate direct quotes from your manuscript), or elaborate on this item by providing additional information not in the ms, or briefly explain why the item is not applicable/relevant for your study

An attrition diagram is not applicable, because "nearly all patients completed the 90-day observation period (Group A, 104 [97.2%]; Group B, 103 [99%]) (Figure 2)."

14a) Dates defining the periods of recruitment and follow-up

Does your paper address CONSORT subitem 14a? *

Copy and paste relevant sections from the manuscript (include quotes in quotation marks "like this" to indicate direct quotes from your manuscript), or elaborate on this item by providing additional information not in the ms, or briefly explain why the item is not applicable/relevant for your study

The study was initiated and first patient enrolled on December 1, 2014 and the last patient completed on September 9, 2015.

14a-i) Indicate if critical "secular events" fell into the study period

Indicate if critical "secular events" fell into the study period, e.g., significant changes in Internet resources available or "changes in computer hardware or Internet delivery resources"

	1	2	3	4	5	
subitem not at all important	○	○	●	○	○	essential

Does your paper address subitem 14a-i?

Copy and paste relevant sections from the manuscript (include quotes in quotation marks "like this" to indicate direct quotes from your manuscript), or elaborate on this item by providing additional information not in the ms, or briefly explain why the item is not applicable/relevant for your study

Not applicable for this study, no critical "secular events" fell within the study period.

14b) Why the trial ended or was stopped (early)

Does your paper address CONSORT subitem 14b? *

Copy and paste relevant sections from the manuscript (include quotes in quotation marks "like this" to indicate direct quotes from your manuscript), or elaborate on this item by providing additional information not in the ms, or briefly explain why the item is not applicable/relevant for your study

Not applicable, the trial was not stopped early.

15) A table showing baseline demographic and clinical characteristics for each group

NPT: When applicable, a description of care providers (case volume, qualification, expertise, etc.) and centers (volume) in each group

Does your paper address CONSORT subitem 15? *

Copy and paste relevant sections from the manuscript (include quotes in quotation marks "like this" to indicate direct quotes from your manuscript), or elaborate on this item by providing additional information not in the ms, or briefly explain why the item is not applicable/relevant for your study

Please see Table 1 in the manuscript, patient demographics and baseline characteristics.

15-i) Report demographics associated with digital divide issues

In ehealth trials it is particularly important to report demographics associated with digital divide issues, such as age, education, gender, social-economic status, computer/Internet/ehealth literacy of the participants, if known.

	1	2	3	4	5	
subitem not at all important	○	●	○	○	○	essential

Does your paper address subitem 15-i? *

Copy and paste relevant sections from the manuscript (include quotes in quotation marks "like this" to indicate direct quotes from your manuscript), or elaborate on this item by providing additional information not in the ms, or briefly explain why the item is not applicable/relevant for your study

Please see Table 1 in the manuscript, patient demographics and baseline characteristics.

16) For each group, number of participants (denominator) included in each analysis and whether the analysis was by original assigned groups

16-i) Report multiple "denominators" and provide definitions

Report multiple "denominators" and provide definitions: Report N's (and effect sizes) "across a range of study participation [and use] thresholds" [1], e.g., N exposed, N consented, N used more than x times, N used more than y weeks, N participants "used" the intervention/comparator at specific pre-defined time

points of interest (in absolute and relative numbers per group). Always clearly define "use" of the intervention.

	1	2	3	4	5	
subitem not at all important	○	○	●	○	○	essential

Does your paper address subitem 16-i? *

Copy and paste relevant sections from the manuscript (include quotes in quotation marks "like this" to indicate direct quotes from your manuscript), or elaborate on this item by providing additional information not in the ms, or briefly explain why the item is not applicable/relevant for your study

N for each group in each analysis is presented in the figures in the manuscript.

16-ii) Primary analysis should be intent-to-treat

Primary analysis should be intent-to-treat, secondary analyses could include comparing only "users", with the appropriate caveats that this is no longer a randomized sample (see 18-i).

	1	2	3	4	5	
subitem not at all important	○	○	●	○	○	essential

Does your paper address subitem 16-ii?

Copy and paste relevant sections from the manuscript (include quotes in quotation marks "like this" to indicate direct quotes from your manuscript), or elaborate on this item by providing additional information not in the ms, or briefly explain why the item is not applicable/relevant for your study

Because this was an open-label study, "All efficacy outcomes were analyzed in the modified intent-to-treat population, which included all randomized patients who had both a baseline value and an on-treatment value for the primary endpoint on or after day 30."

Revised

17a) For each primary and secondary outcome, results for each group, and the estimated effect size and its precision (such as 95% confidence interval)

Does your paper address CONSORT subitem 17a? *

Copy and paste relevant sections from the manuscript (include quotes in quotation marks "like this" to indicate direct quotes from your manuscript), or elaborate on this item by providing additional information not in the ms, or briefly explain why the item is not applicable/relevant for your study

Efficacy results are presented as least squares means with 95% confidence intervals, for example, "Improvement in mobility at day 90 was significantly greater in Group A than in Group B. LS mean change in number of steps/day was 1199 vs 467, a mean difference of 732 steps (95% CI [127,1337]; P = .03)."

17a-i) Presentation of process outcomes such as metrics of use and intensity of use

In addition to primary/secondary (clinical) outcomes, the presentation of process outcomes such as metrics of use and intensity of use (dose, exposure) and their operational definitions is critical. This does not only refer to metrics of attrition (13-b) (often a binary variable), but also to more continuous exposure metrics such as "average session length". These must be accompanied by a technical description how a metric like a "session" is defined (e.g., timeout after idle time) [1] (report under item 6a).

	1	2	3	4	5	
subitem not at all important	○	○	●	○	○	essential

Does your paper address subitem 17a-i?

Copy and paste relevant sections from the manuscript (include quotes in quotation marks "like this" to indicate direct quotes from your manuscript), or elaborate on this item by providing additional information not in the ms, or briefly explain why the item is not applicable/relevant for your study

Not applicable for this study. Metrics such as average session length were not measured.

17b) For binary outcomes, presentation of both absolute and relative effect sizes is recommended

Does your paper address CONSORT subitem 17b? *

Copy and paste relevant sections from the manuscript (include quotes in quotation marks "like this" to indicate direct quotes from your manuscript), or elaborate on this item by providing additional information not in the ms, or briefly explain why the item is not applicable/relevant for your study

Binary outcomes are not applicable for this study.

18) Results of any other analyses performed, including subgroup analyses and adjusted analyses, distinguishing pre-specified from exploratory

Does your paper address CONSORT subitem 18? *

Copy and paste relevant sections from the manuscript (include quotes in quotation marks "like this" to indicate direct quotes from your manuscript), or elaborate on this item by providing additional information not in the ms, or briefly explain why the item is not applicable/relevant for your study

Not applicable, only primary and secondary outcomes were analyzed and presented in this manuscript.

18-i) Subgroup analysis of comparing only users

A subgroup analysis of comparing only users is not uncommon in ehealth trials, but if done, it must be stressed that this is a self-selected sample and no longer an unbiased sample from a randomized trial (see 16-iii).

1 2 3 4 5

subitem not at all important ○ ○ ○ ○ ○ essential

Does your paper address subitem 18-i?

Copy and paste relevant sections from the manuscript (include quotes in quotation marks "like this" to indicate direct quotes from your manuscript), or elaborate on this item by providing additional information not in the ms, or briefly explain why the item is not applicable/relevant for your study

Not applicable for this study, subgroup analysis comparing only users was not done.

19) All important harms or unintended effects in each group

(for specific guidance see CONSORT for harms)

Does your paper address CONSORT subitem 19? *

Copy and paste relevant sections from the manuscript (include quotes in quotation marks "like this" to indicate direct quotes from your manuscript), or elaborate on this item by providing additional information not in the ms, or briefly explain why the item is not applicable/relevant for your study

"The occurrence of TEAEs was similar in the 2 groups (Table 2) with no new safety signals noted. Arthralgia (Group A, 8 [7.5%]; Group B, 12 [11.5%]) and upper respiratory tract infection (Group A, 7 [6.5%]; Group B, 2 [1.9%]) were the 2 most commonly reported TEAEs among patients. No major AEs related to the device or protocol occurred." Also see Table 2 in the manuscript.

19-i) Include privacy breaches, technical problems

Include privacy breaches, technical problems. This does not only include physical "harm" to participants, but also incidents such as perceived or real privacy breaches [1], technical problems, and other unexpected/unintended incidents. "Unintended effects" also includes unintended positive effects [2].

1 2 3 4 5

subitem not at all important ◯ ◯ ◯ ◯ ◯ essential

Does your paper address subitem 19-i?

Copy and paste relevant sections from the manuscript (include quotes in quotation marks "like this" to indicate direct quotes from your manuscript), or elaborate on this item by providing additional information not in the ms, or briefly explain why the item is not applicable/relevant for your study

Not applicable, no privacy breaches occurred during this study.

19-ii) Include qualitative feedback from participants or observations from staff/researchers

Include qualitative feedback from participants or observations from staff/researchers, if available, on strengths and shortcomings of the application, especially if they point to unintended/unexpected effects or uses. This includes (if available) reasons for why people did or did not use the application as intended by the developers.

1 2 3 4 5

subitem not at all important ◯ ◯ ◯ ◯ ◯ essential

Does your paper address subitem 19-ii?

Copy and paste relevant sections from the manuscript (include quotes in quotation marks "like this" to indicate direct quotes from your manuscript), or elaborate on this item by providing additional information not in the ms, or briefly explain why the item is not applicable/relevant for your study

"A greater number of Group A patients (68, 65.4%) reported they would be likely or very likely to use the devices compared with patients (36, 34.6%) who reported that they would be somewhat likely or not at all likely to do so (Figure 5). Seventy-six physicians answered surveys for 104 patients; 70 of 104 (67.3%) physician surveys reported physicians to be likely or very likely to recommend use of the devices versus 34 (32.7%) surveys reporting physicians to be who would only be somewhat likely or not at all likely to recommend their use (Figure 5)."

Revised

DISCUSSION

22) Interpretation consistent with results, balancing benefits and harms, and considering other relevant evidence

NPT: In addition, take into account the choice of the comparator, lack of or partial blinding, and unequal expertise of care providers or centers in each group

22-i) Restate study questions and summarize the answers suggested by the data, starting with primary outcomes and process outcomes (use)

Restate study questions and summarize the answers suggested by the data, starting with primary outcomes and process outcomes (use).

	1	2	3	4	5	
subitem not at all important	○	○	○	●	○	essential

Does your paper address subitem 22-i? *

Copy and paste relevant sections from the manuscript (include quotes in quotation marks "like this" to indicate direct quotes from your manuscript), or elaborate on this item by providing additional information not in the ms, or briefly explain why the item is not applicable/relevant for your study

"Walking has been shown to have a beneficial effect on symptoms and decreases risk of functional limitations in patients with knee OA [19,20]; however, 66% of patients with arthritis reported walking for fewer than 90 minutes per week [25]. The current study showed that, in patients treated with hylan G-F 20, those also using the OA GO app significantly improved their mobility with an increase in over twice as many steps per day and over 3 times the percentage change in steps per day compared with patients not using this motivating app."

22-ii) Highlight unanswered new questions, suggest future research

Highlight unanswered new questions, suggest future research.

	1	2	3	4	5	
subitem not at all important	○	○	○	●	○	essential

Does your paper address subitem 22-ii?

Copy and paste relevant sections from the manuscript (include quotes in quotation marks "like this" to indicate direct quotes from your manuscript), or elaborate on this item by providing additional information

not in the ms, or briefly explain why the item is not applicable/relevant for your study

"These results also highlight the amenability of patients and physicians to using mobile health technology in the treatment of OA and suggest further investigation is warranted."

20) Trial limitations, addressing sources of potential bias, imprecision, and, if relevant, multiplicity of analyses

20-i) Typical limitations in ehealth trials

Typical limitations in ehealth trials: Participants in ehealth trials are rarely blinded. Ehealth trials often look at a multiplicity of outcomes, increasing risk for a Type I error. Discuss biases due to non-use of the intervention/usability issues, biases through informed consent procedures, unexpected events.

	1	2	3	4	5	
subitem not at all important	○	○	○	○	●	essential

Does your paper address subitem 20-i? *

Copy and paste relevant sections from the manuscript (include quotes in quotation marks "like this" to indicate direct quotes from your manuscript), or elaborate on this item by providing additi[illegible] not in the ms, or briefly explain why the item is not applicable/relevant for your study

Revised

"The patients studied may not be fully representative of the general OA population with respect to gender distribution (women are at higher risk for knee OA than men [42]), and activity levels as inclusion/exclusion criteria were such that patients with low levels (<500 steps/day) and high levels (>8000 steps/day) of baseline activity were not eligible for enrollment. Patients with a BMI >35 were also excluded from the study. In addition, the Kellgren -Lawrence grade of patients' knee OA was not collected, so data could not be stratified and analyzed in relation to radiographic disease severity, which might have been helpful in clarifying some of the study results such as the PAM-13 scores. The patient and physician satisfaction survey collected important feedback but is not an externally validated instrument. Moreover, the study was short-term, having been conducted over 90 days. Finally, the study did not include an arm in which patients used the motivating app and did not receive an injection, nor did it include an arm in which patients used the motivating app and received a saline injection (often designated as placebo)."

21) Generalisability (external validity, applicability) of the trial findings

NPT: External validity of the trial findings according to the intervention, comparators, patients, and care providers or centers involved in the trial

21-i) Generalizability to other populations

Generalizability to other populations: In particular, discuss generalizability to a general Internet population, outside of a RCT setting, and general patient population, including applicability of the study results for other organizations

	1	2	3	4	5	
subitem not at all important	○	○	○	●	○	essential

Revised

Does your paper address subitem 21-i?

"**Implication of Current Study Findings**
Use of the OA GO app provides direct feedback on mobility function and pain (whereby steps and mobility become surrogates for pain and function, assuming there are no other reasons for decreased activity), which should give a more valid and reliable report of pain over time as it does not rely on patient recall of pain experienced. In contrast, visual analog scales [35] and the Western Ontario and McMaster Universities Osteoarthritis Index (used to assess pain, stiffness, and physical function over a specific time frame [ie, the last 48 hours] in clinical trials [36]) are both subjective measures that rely on patients thinking back to how they perceived they were feeling. Furthermore, symptoms of OA are often variable depending on a number of factors, including patients' activity level [7] and changes in the weather [37], suggesting that pain should be assessed in the context of ongoing physical activity levels. This technology, therefore, is an objective measure of functional improvement over time resulting from clinical interventions provided to patients, making it not only a motivating device for patients, but also an important research tool."

21-ii) Discuss if there were elements in the RCT that would be different in a routine application setting

Discuss if there were elements in the RCT that would be different in a routine application setting (e.g., prompts/reminders, more human involvement, training sessions or other co-interventions) and what impact the omission of these elements could have on use, adoption, or outcomes if the intervention is applied outside of a RCT setting.

	1	2	3	4	5	
subitem not at all important	○	○	●	○	○	essential

Does your paper address subitem 21-ii?

Copy and paste relevant sections from the manuscript (include quotes in quotation marks "like this" to indicate direct quotes from your manuscript), or elaborate on this item by providing additional information not in the ms, or briefly explain why the item is not applicable/relevant for your study

Not applicable for this study, wearable activity monitor and smartphone app were used in real-world settings.

OTHER INFORMATION

23) Registration number and name of trial registry

Does your paper address CONSORT subitem 23? *

Copy and paste relevant sections from the manuscript (include quotes in quotation marks "like this" to indicate direct quotes from your manuscript), or elaborate on this item by providing additional information not in the ms, or briefly explain why the item is not applicable/relevant for your study

"After working with the FDA to initiate registration of the trial, the sponsor deemed the study not eligible to be posted on ClinicalTrials.gov considering that clinical studies using devices whereby the primary outcome measure relates solely to feasibility, and not to health outcomes, are excluded from registration. The FDA concurred there was not a requirement to register the trial at that time."

24) Where the full trial protocol can be accessed, if available

Does your paper address CONSORT subitem 24? *

Cite a Multimedia Appendix, other reference, or copy and paste relevant sections from the manuscript (include quotes in quotation marks "like this" to indicate direct quotes from your manuscript), or elaborate on this item by providing additional information not in the ms, or briefly explain why the item is not applicable/relevant for your study

The trial protocol is not publicly available.

25) Sources of funding and other support (such as supply of drugs), role of funders

Does your paper address CONSORT subitem 25? *

Copy and paste relevant sections from the manuscript (include quotes in quotation marks "like this" to indicate direct quotes from your manuscript), or elaborate on this item by providing additional information not in the ms, or briefly explain why the item is not applicable/relevant for your study

"Sanofi Biosurgery, LLC funded the study."

X27) Conflicts of Interest (not a CONSORT item)

X27-i) State the relation of the study team towards the system being evaluated

In addition to the usual declaration of interests (financial or otherwise), also state the relation of the study team towards the system being evaluated, i.e., state if the authors/evaluators are distinct from or identical with the developers/sponsors of the intervention.

	1	2	3	4	5	
subitem not at all important	○	○	○	●	○	essential

Does your paper address subitem X27-i?

Copy and paste relevant sections from the manuscript (include quotes in quotation marks "like this" to indicate direct quotes from your manuscript), or elaborate on this item by providing additional information

not in the ms, or briefly explain why the item is not applicable/relevant for your study

Investigators did not develop the smartphone application.

About the CONSORT EHEALTH checklist

As a result of using this checklist, did you make changes in your manuscript? *

- yes, major changes
- yes, minor changes
- no

Revised

What were the most important changes you made as a result of using this checklist?

Increased details on population studied and randomization procedure.

How much time did you spend on going through the checklist INCLUDING making changes in your manuscript *

Six hours.

As a result of using this checklist, do you think your manuscript has improved? *

- yes
- no
- Other: Some additional details were added to the manuscript.

Would you like to become involved in the CONSORT EHEALTH group?

This would involve for example becoming involved in participating in a workshop and writing an "Explanation and Elaboration" document

- yes
- no
- Other:

Any other comments or questions on CONSORT EHEALTH

STOP - Save this form as PDF before you click submit

To generate a record that you filled in this form, we recommend to generate a PDF of this page (on a Mac, simply select "print" and then select "print as PDF") before you submit it.

When you submit your (revised) paper to JMIR, please upload the PDF as supplementary file.

Don't worry if some text in the textboxes is cut off, as we still have the complete information in our database. Thank you!

Final step: Click submit !

Click submit so we have your answers in our database!

Submit

Never submit passwords through Google Forms.

Powered by

This content is neither created nor endorsed by Google.

Report Abuse - Terms of Service - Additional Terms
